# As predicted and more: modulated channel occupation in YZn_5+*x*_

**DOI:** 10.1107/S2052520623005292

**Published:** 2023-07-07

**Authors:** Rie T. Fredrickson, Daniel C. Fredrickson

**Affiliations:** a Department of Chemistry, University of Wisconsin-Madison, 1101 University Avenue, Madison, WI 53706, USA; Academy of Sciences of the Czech Republic, Czech Republic

**Keywords:** aperiodic crystals, crystal structure prediction, intermetallic phases

## Abstract

Like many complex intermetallic phases, the crystal structures of REZn_5+*x*_ compounds (RE = lanthanide or Group 3 element) based on the EuMg_5_ type have gradually unfolded. The original reports described a complex hexagonal structure with an unusual combination of tetrahedrally close-packed regions and open spaces, as well as observations of superstructure reflections. More recently, we reinvestigated the structure of YZn_5_, reclassifying it as the EuMg_5+*x*_-type compound YZn_5+*x*_ (*x* ≃ 0.2), in which disordered channels run along *c* through the spaces formerly considered open. In addition, DFT-chemical pressure (DFT-CP) analysis of ordered models of YZn_5+*x*_ highlighted paths for communication between neighboring channels setting the stage for superstructure formation. Herein, the experimental elucidation of this effect is presented with the synthesis and structure determination of a modulated form of YZn_5+*x*_. By slow-cooling samples of YZn_5+*x*_ from the annealing temperature, crystals were obtained that exhibit satellite reflections with the modulation wavevector **q** =  a* +  b* + 0.3041c*. Structure solution and refinement using a (3+1)D model in superspace group *P* 31*c*(  σ_3_)00*s* reveals incommensurate order in the structure’s channels. Here, two Zn sites associated with the channels are present, each with discontinuous atomic domains that are slanted in the *x*_3_ *x*_4_ plane. Their slanting corresponds to adjustments along the *c* axis for the presence or absence of close neighbors along that axis, while the occupation patterns of neighboring channels are shifted by  of the modulation period. These features follow earlier predictions from CP analysis, highlighting how this approach can be used predictively in search of new phenomena.

## Abstract 2

The superspace approach is used to determine the structure of a modulated form of YZn_5+*x*_ (*x* = 0.217), which is an ordered variant of the previously described EuMg_5+*x*_-type structure, with its channels containing disordered Zn atoms. The details of the pattern align closely with earlier predictions based on DFT-chemical pressure analysis of ordered models.

## 1. Introduction

When encountering disorder in a newly solved crystal structure, one faces several questions. Could an ordered superstructure be obtainable under different conditions or through variations in the crystallization or synthesis methods (Etzkorn *et al.*, 2007 ▸; Lidin *et al.*, 2009 ▸; Okamoto *et al.*, 2017 ▸; Becker & Leineweber, 2018 ▸)? Or may a superstructure be present, but obscured by the balanced twinning of domains related by pseudosymmetry (Feuerbacher *et al.*, 2007 ▸)? Does the randomness suggested by the crystallographic model belie local order that is just not pronounced enough to be detectable as diffuse scattering in the data available (Frey, 1995 ▸; Larsson *et al.*, 1996 ▸)? Together, such possibilities relate to the origins of the apparent disorder in frustration or near degeneracy (Keen & Goodwin, 2015 ▸), and the potential ways in which the occupancies or positions of atoms in one region of disorder impacts the preferences of others. Over the past ten years, the DFT-chemical pressure (CP) analysis (Hilleke & Fredrickson, 2018 ▸; Lu *et al.*, 2021 ▸) has emerged as a theoretical approach to analyze these effects, as illustrated by its ability to reveal paths of soft atomic motion underlying the channel-like structures of the Nowotny Chimney Ladders (Lu & Fredrickson, 2019 ▸) and Fe_2_Al_5_ (Vinokur *et al.*, 2019 ▸), as well as the packing issues faced by the disordered polyhedra in the quasicrystal approximant CaCd_6_ (Berns & Fredrickson, 2013 ▸) and Fe_14_Pd_17_Al_69_ (Peterson *et al.*, 2020 ▸). More recently, this approach has been applied in a predictive fashion, in which CP analysis on models of the disordered EuMg_5+*x*_-type compound YZn_5+*x*_ (*x* ≃ 0.2) pointed to a potential superstructure (Fredrickson *et al.*, 2022 ▸), a proposal that we herein experimentally investigate.

**Figure 1:**
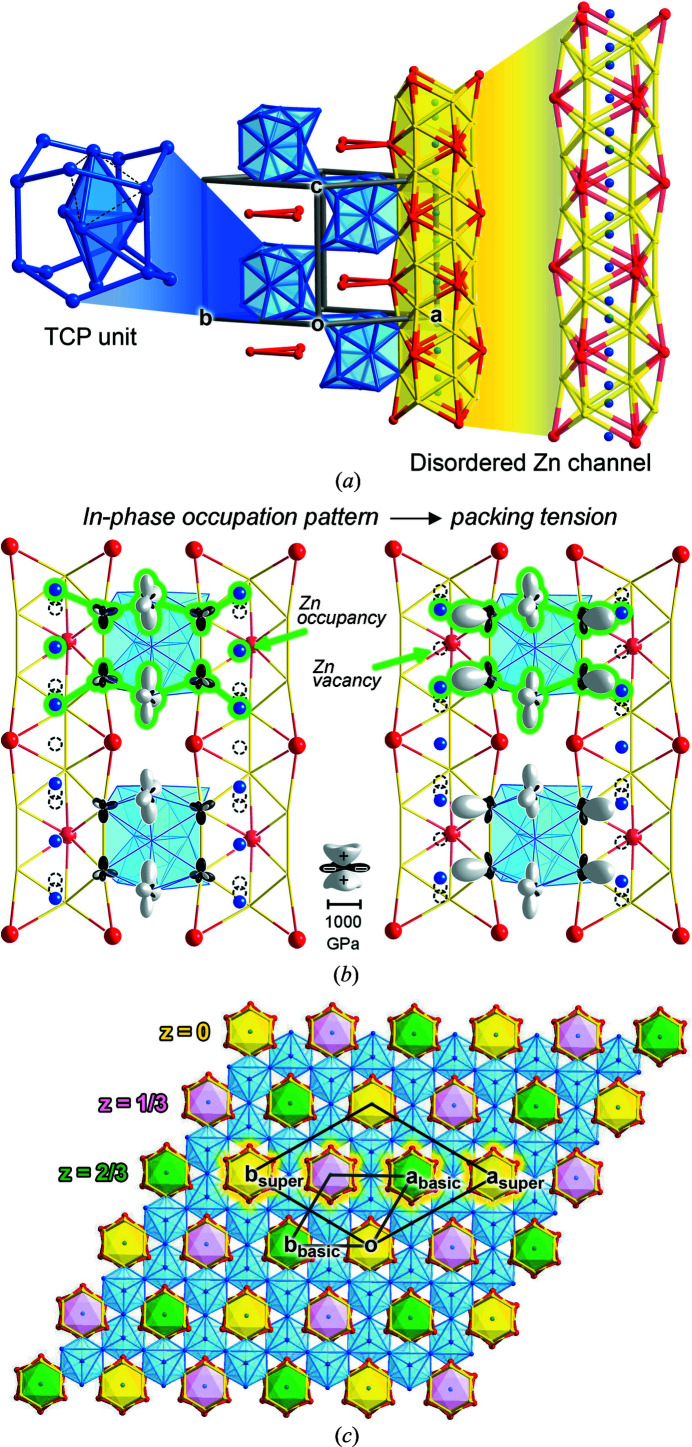
The disordered form of YZn_5+*x*_ (*x* ≃ 0.2) and its predicted superstructure from CP analysis. (*a*) The disordered YZn_5+*x*_ structure is constructed with tetrahedrally close packed (tcp) units, Y triangles, and Y–Zn channels (yellow/red) that contain disordered Zn atoms (blue). (*b*) Selected CP features calculated for ordered models of YZn_5+*x*_, shown for points where neighboring Zn channels are bridged by tcp units, with the in-plane Zn positions in the channel being filled (left) or vacant (right). Black lobes correspond to directions of negative CP (where contraction is preferred), while white lobes indicate positive CP (where expansion of the structure would be favorable). (*c*) The 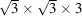 superstructure predicted from the CP analysis, viewed down [001] with its basic cell outlined. Three colors are used for the channels to indicate the 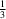 **c**_super_ shifts for the Zn occupation patterns between neighboring columns: yellow for Δ*z* = 0, pink for Δ*z* = 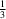 , and green for Δ*z* = 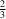 .

Several REZn_5_ compounds (RE = lanthanide or Group 3 transition metal) were originally reported by Fornasini and coworkers as adopting the EuMg_5_ type (Mühlpfordt, 1970 ▸), with the observation that some crystals exhibited superstructure reflections corresponding to 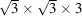 or 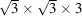 × 6 supercells (Bruzzone *et al.*, 1970 ▸; Fornasini, 1971 ▸). Intrigued by the structural relationship of these phases to the family of CP-driven variations of the CaCu_5_ type (Berns & Fredrickson, 2014 ▸), as well as unusual open void spaces that appear in them, we recently reinvestigated the crystal structure of YZn_5_ as a representative example (Fredrickson *et al.*, 2022 ▸). Our structure solution revealed columns of disordered Zn atoms running along the hexagonal **c** axis [Fig. 1 ▸(*a*)], placing this compound in the EuMg_5+*x*_ type (Erassme & Lueken, 1987 ▸; Mühlpfordt, 1997 ▸), a disordered variant of the EuMg_5_ type adopted not only in RE–Mg and RE–Zn systems but also observed in Ni- and Al-based intermetallics (Gladyshevskii *et al.*, 1993 ▸; Bohdana *et al.*, 2020 ▸).

While the X-ray diffraction patterns of these crystals showed no indication of a superstructure, DFT-CP analysis on a series of ordered models pointed to a mechanism for the occupation pattern of one channel to influence those of its surroundings [Fig. 1 ▸(*b*)]: neighboring channels in the *ab* plane are bridged through tetrahedrally close-packed (tcp) units (blue), which are subject to different CP effects depending on whether the adjacent Zn sites in the channel are occupied or vacant. In both cases, the occupation or vacancy of the channel atom on both sides of the tcp unit leads to an accumulation of stress, which could be relieved through a staggering of the occupation pattern of neighboring columns. Based on this scheme, we proposed a superstructure pattern [Fig. 1 ▸(*c*)] in which Zn atoms are distributed in an ordered fashion over two basic Zn positions along each channel to give rise to a tripling of the **c** repeat vector, with the patterns of neighboring channels being shifted relative to each other by one subcell.

In this article, we experimentally demonstrate and elucidate the occurrence of channel ordering in YZn_5+*x*_. Through a simple change in the temperature treatment in the synthetic procedure—allowing the reaction to slowly cool to ambient conditions from the annealing temperature (as opposed to quenching them in ice water)—crystals are obtained that exhibit both sharp diffraction peaks for the EuMg_5+*x*_-type basic cell and additional reflections at intermediate points in reciprocal space. While the latter correspond approximately to the expected 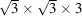 supercell, their small number and relative weakness make solution as a conventional structure challenging. As we will see below, the structure solution and refinement proceed much more smoothly using the (3+1)D superspace approach (Janner & Janssen, 1980*a* ▸,*b* ▸; van Smaalen, 2007 ▸), in which the additional reflections are indexed as satellites with **q**-vector **q** = σ_1_ a* + σ_2_ b* + σ_3_ c* = 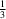 a* + 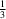 b* + 0.3041c*, and the structure is modeled as periodic in (3+1)D space with the additional dimension corresponding to the phase of a modulation function. The emerging crystal structure model confirms the general ordering pattern predicted by CP analysis, highlighting the predictive potential of CP-based models of structural effects.

## 2. Experimental

### 2.1. Synthesis

In our synthetic investigation of the Y–Zn system, elemental Y (Strem Chemicals, 99.9%) and Zn (Alfa Aesar, 99.9%) were used as starting materials. The elements were weighed out in the stoichiometric molar ratio of Y:Zn = 1:5 in an Ar-filled glove box, pressed into a pellet, and then placed into a fused silica tube, which was then evacuated and sealed. The sample was heated to 1100°C for 24 h to achieve a homogeneous melt, then annealed at 500°C for 168 h to allow for crystal growth, and finally cooled slowly to ambient temperature at a rate of 10°C h^−1^.

### 2.2. Single crystal X-ray diffraction analysis

Single crystal X-ray diffraction data for YZn_5+*x*_ (*x* = 0.217) were collected on an Oxford Diffraction Xcalibur E diffractometer using graphite monochromated Mo *K*α radiation (λ = 0.71073 Å) at ambient temperature. The collection and processing of the data set were performed using the *CrysAlis Pro* (v. 171.42.49; Rigaku Oxford Diffraction, 2022 ▸) software. The structure was solved with the charge flipping algorithm (Oszlányi & Sütő, 2004 ▸, 2005 ▸; Palatinus, 2004 ▸) using the program *SUPERFLIP* (Palatinus & Chapuis, 2007 ▸) and refined on *F* ^2^ using the program *JANA2006* (Petříček *et al.*, 2014 ▸). Further details regarding the refinements are given in Table 1 ▸ and the supporting information. The refined atomic coordinates and atomic displacement parameters for the basic structure are given in Tables 2 ▸ and 3 ▸, respectively.

### 2.3. Powder X-ray diffraction analysis

Phase analysis of the sample was performed using powder X-ray diffraction measurements. The sample was ground to a fine powder and placed on a zero-background plate. Diffraction intensities were measured on a Bruker D8 Advance Powder Diffractometer fitted with a LYNXEYE detector, using Cu *K*α radiation (λ = 1.5418 Å) at ambient temperature. An exposure time of 1.0 s per 0.010° increment was used over the 2θ range 30–80°.

### 2.4. Wavelength-dispersive X-ray spectroscopy

To determine the elemental composition of YZn_5+*x*_, wavelength dispersive X-ray spectroscopy (WDS) was performed on a sample whose powder X-ray diffraction pattern showed this to be the major phase. To prepare the sample for WDS measurements, a small amount of material was suspended in a conductive ep­oxy at one end of a short segment of an aluminium tube. Once the ep­oxy had hardened, the sample was ground down to produce a flat surface and then polished to reduce the number of surface scratches using a diamond lapping film (Precision Surface International Inc., 0.5 µm). Finally, the sample was carbon-coated. WDS measurements guided by back-scattered electron images were taken with a Cameca SX-Five FE-EMPA Microprobe, using Y_2_O_3_, elemental Zn, elemental Si, and ZnO as standards for the Y *L*α, Zn *K*α, Si *K*α, and O *K*α transitions, respectively.

## 3. Results and discussion

### 3.1. Synthesis of incommensurately modulated YZn_5+*x*_ crystals

In our earlier investigation of the disordered YZn_5+*x*_ (*x* ≃ 0.2) structure, we uncovered channels of disordered Zn atoms running along the **c** axis, making this compound a member of the EuMg_5+*x*_ type (Fredrickson *et al.*, 2022 ▸). We also noted, however, that a mechanism exists for the communication between the occupants of neighboring channels, opening a path to a potential ordered superstructure. Based on these interactions, we proposed a model for a 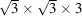 superstructure. In our attempt to synthetically realize this superstructure, we carried out a modified version of our synthetic procedure. As before we pressed mixtures of the elements into pellets, brought them to a melt at 1100°C for 24 h and held them at 500°C for one week for crystal growth. This time, however, rather than quenching the samples in ice water, we introduced a controlled cooling step (10°C h^−1^) to encourage superstructure formation. The reaction resulted in relatively large, well defined crystals, particularly in comparison to the quenched products from our previous syntheses containing the disordered YZn_5+*x*_ crystals. The crystals are metallic gray in color, with smooth surfaces, and are easy to grind into powders. In addition, the crystals exhibit high stability in air with no visible decomposition.

**Figure 2:**
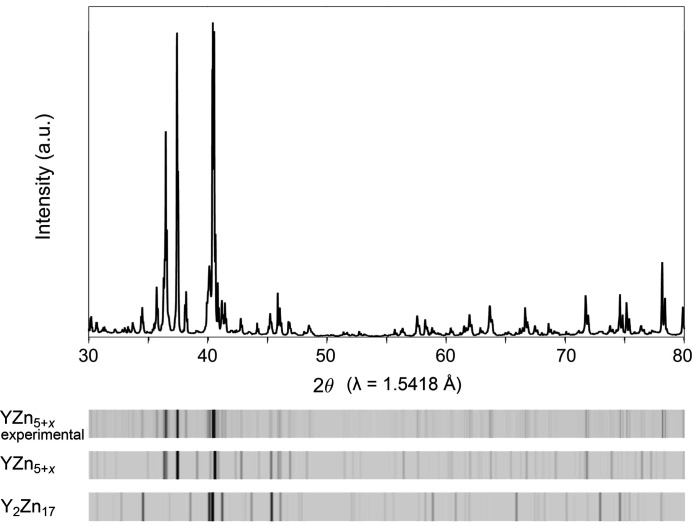
Powder X-ray diffraction pattern of the modulated form of YZn_5+*x*_ (radiation: Cu *K*α). Top: the experimental data plotted in a 2θ versus intensity form. Bottom: filmstrip style representation of the experimental data along with the simulated patterns for YZn_5+*x*_ and Y_2_Zn_17_.

The powder X-ray diffraction data collected for a sample from which we picked the single crystal of YZn_5+*x*_ (*x* = 0.217) is presented in Fig. 2 ▸. All the major peaks are indexed to the YZn_5+*x*_ structure by reference to the single crystal data described below. Some minor peaks are assigned to a secondary phase, Y_2_Zn_17_, as well as others that appear to arise from an unknown, third phase. These results are consistent with metallographic analysis of a polished sample with back-scattered electron (BSE) imaging and WDS. BSE revealed two prominent phases in the sample: one appearing lighter, the other darker. The composition for the first of these was determined to be YZn_5.26 (3)_ from an average over 43 points, which corresponds closely with that expected for YZn_5+*x*_. The second phase has the composition Y_2_Zn_16.81 (8)_ (average over 30 points); this is assigned as the compound Y_2_Zn_17_. Needle-like domains of a third, minor phase with approximate composition YZn_2.2_Si_0.6_ were also occasionally observed, which apparently results from a reaction with SiO_2_ from the fused silica ampoule. Altogether, though, the powder X-ray diffraction pattern and WDS analysis indicates the YZn_5+*x*_ is a major phase in the reaction product.

### 3.2. Analysis of diffraction pattern and symmetry

**Figure 3:**
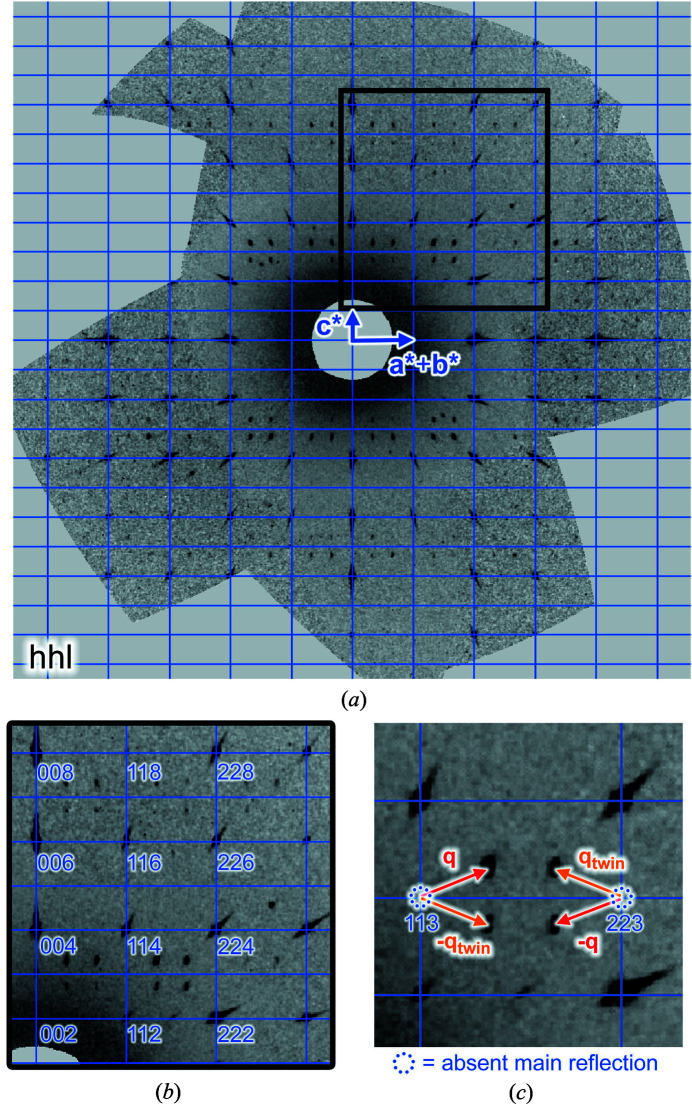
Diffraction pattern of a YZn_5+*x*_ (*x* = 0.217) crystal. (*a*) The satellite reflections exhibited along with the strong main reflections in the *hhl* plane. (*b*) Enlarged area, with the indices of the main reflections. (*c*) Indexation of the satellite reflections with the modulation wavevector (**q** = 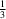 a* + 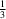 b* + 0.3041c*) (red) and the corresponding vector for a twin domain (orange). Blue dotted circles denote absent main reflections.

Crystals picked from these reaction products exhibit more complex diffraction patterns [Fig. 3 ▸(*a*)] than those obtained from quenching the samples from the annealing temperature. The strongest reflections can be indexed to a hexagonal cell, corresponding to the EuMg_5+*x*_-type basic structure, with dimensions *a* = 8.8811 (1) Å and *c* = 9.2057 (1) Å. Additional weaker reflections are present in the spaces between these main peaks, as is evident in the reconstruction of the (*hhl*) layer of reciprocal space (Fig. 3 ▸). These new reflections correspond approximately to a 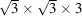 supercell, but the distribution of their intensities is highly organized. They trace out rectangles, with the closest reciprocal lattice points of the basic cell being the systematically absent *l* = odd positions (*e.g. hhl* = 113, 223 *etc*.). A careful investigation for the positions of the superstructure reflections reveals that they are all separated from an absent main reflection by one of two vectors: **q** = 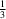 a* + 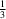 b* + 0.3041c* or **q**′ = − 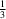 a*  − 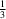 b* + 0.3041c*, as shown in Figs. 3 ▸(*b*) and 3 ▸(*c*), each of which creates a pattern with trigonal symmetry. No other reflections associated with a supercell are evident, suggesting that a superspace approach that considers the additional peaks to arise from modulations will be the most efficient route to solving the structure. In particular, a (3+1)D model (Smaalen, 2007 ▸) can be created when we assign **q** and **q**′ as belonging to separate twin domains, which are related by a sixfold rotation around c*, in-line with the hexagonal symmetry of the EuMg_5+*x*_ type basic structure. While there are several multiples of 60° that could be used to generate these twin domains, the threefold symmetry of the individuals will lead to only two symmetry-distinct crystal orientations.

In this approach, the experimental diffraction pattern is interpreted as the projection on 3D of a (3+1)D array of reflections, with the satellites having a component along the fourth dimension. The corresponding real space construction is a periodic structure in (3+1)D from which the physical incommensurately modulated structure is obtained by taking 3D cross-sections. The direction perpendicular to the physical cross-section is given by the **a** *_s_*_4_ axis, which represents the superspace lattice vector for the modulation function, while the **a** *_s_*_1_, **a** *_s_*_2_ and **a** *_s_*_3_ axes are counterparts to the physical **a**, **b** and **c** axes. They are, however, tilted out of the physical space axes in accordance with how much the phase of the modulation function changes for translations along the **a**, **b** and **c** repeat vectors of the basic cell.

A first step in building such a model is the assignment of the structure’s (3+1)D superspace group. The **q**-vector **q** = 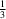 a* + 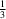 b* + 0.3041c* gives rise to a trigonal arrangement of satellite reflections, which is incompatible with the hexagonal space group *P*6_3_/*mmc* of the EuMg_5+*x*_ type. In terms of potential trigonal subgroups of *P*6_3_/*mmc*, we observe that the systematic absence condition associated with the *c* glide in *P*6_3_/*mmc*, *hhl*: *l* = 2*n_,_* still applies to the main reflections (Fig. 3 ▸), suggesting that the superspace group of the modulated structure is based on the *P*6_3_/*mmc* subgroup 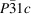 . When we consider the full set of reflections, it is notable that the satellites only appear in the layer when *l* is odd, pointing to the condition *hhlm*: *l*+*m* = 2*n*. In terms of the symmetry operations of the (3+1)D superspace group, this corresponds to the *c* glide operation in 3D space being accompanied by a shift in the phase of the modulation (*x*_4_) by ½. Altogether, these considerations indicate that the highest possible superspace group is *P* 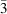 1*c*( 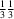 σ_3_)00*s*.

### 3.3. Structure solution and refinement

In the structure solution process, the charge flipping algorithm using the program *Superflip* converged on a (3+1)D electron density corresponding to the experimental diffraction intensities. The automated symmetry search and peak analysis of this electron density map agreed with the assignment of the superspace group as *P* 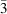 1*c*( 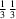 σ_3_)00*s*, and yielded the basic framework of the EuMg_5+*x*_-type basic structure. In addition, two atomic domains associated with the disordered channels in the EuMg_5+*x*_ type were identified, which we label Zn5 and Zn6.

**Figure 4:**
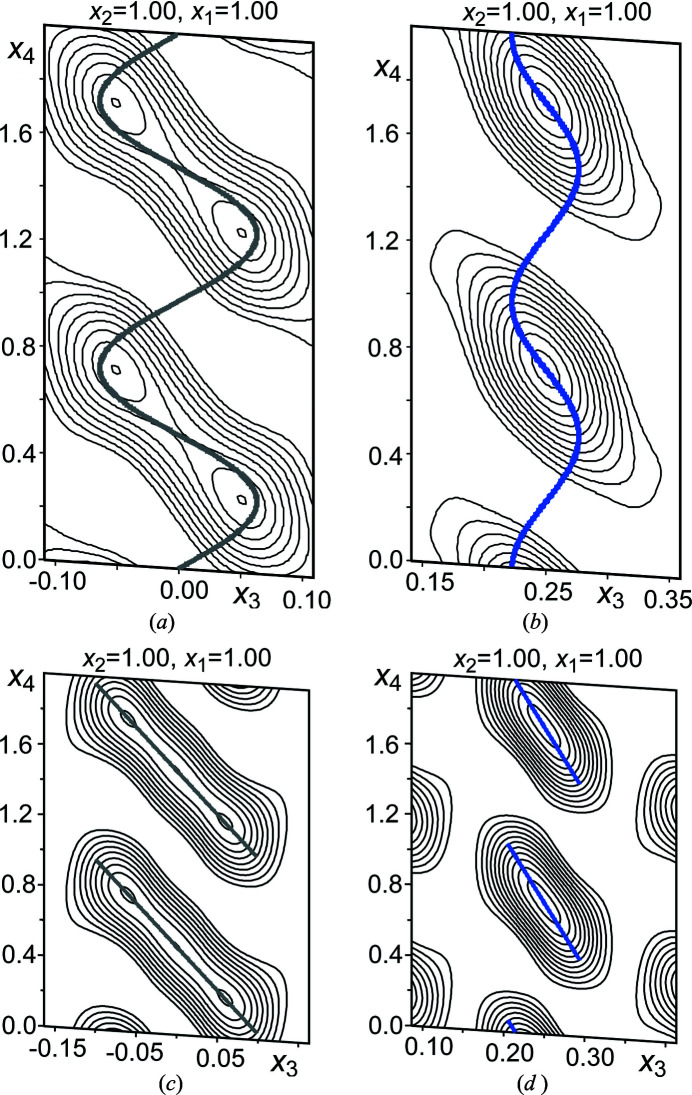
Contour maps of the Fourier electron density for (*x*_3_, *x*_4_) cross-sections through the Zn5 and Zn6 atomic domains. Top: the contours generated from the structure solution for the (*a*) Zn5 and (*b*) Zn6 sites show positionally modulated and discontinuous atomic domains. Bottom: the corresponding electron density maps for the refined model, in which atomic modulation functions with crenel functions are used for the (*c*) Zn5 and (*d*) Zn6 sites. The model positions for Zn5 and Zn6 are shown in gray and blue, respectively.

The electron density features for the Zn5 and Zn6 sites show strong modulations with both occupational and positional components [Figs. 4 ▸(*a*) and 4 ▸(*b*)]. For the Zn5 site, the electron density contours in the *x*_3_ *x*_4_ section trace out nearly linear domains centered at *x*_4_ = 0.5 that are slanted to give a negative slope, and end on discontinuities near *x*_4_ = 0.0 and 1.0. Similar features are found for the Zn6 site, except that its domain is centered at *x*_4_ = 0.75, and its length along *x*_4_ is shorter, with the width Δ*x*_4_ being about 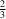 . To model the discontinuous nature of these domains, we used crenel functions (Petříček *et al.*, 2016 ▸) centered at *x*_4_ = 0.5 and *x*_4_ = 0.75 for Zn5 and Zn6 respectively, with the corresponding Δ*x*_4_ values initially being set to 1.0 and 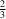 , where the latter is a close rational approximant to ½ + ½σ_3_ (the Δ*x*_4_ width for Zn6 needed to match the ends of the Zn5 and Zn6 domains in the physical cross-sections; see the supporting information). This is convenient as σ_3_ varies from crystal to crystal (in the range of 0.304 to ∼0.331). In addition, first order positional modulations (based on Legendre polynomials tailored to the Δ*x*_4_ width of the domain) were applied to allow the atomic domains to slant in the *x*_3_ *x*_4_ plane.

After setting these modulation functions for the Zn5 and Zn6 sites, in addition to first order harmonic positional waves on the remaining atoms, the refinement led quickly to a drop in the *R* factors for the satellite reflections. However, additional corrections were needed for a successful refinement. First, a twin component was included whose reciprocal lattice is rotated around the *c* axis by 60°, reflecting the hexagonal symmetry of the parent structure. Curiously, though, the twin-fraction invariably refined to approximately 0.75, regardless of which individual is integrated. As the diffraction patterns for the individuals overlap only on the main reflections, this suggested that the main reflections are systematically too strong relative to the satellites. Interpreting this as arising from disordered domains in the crystal, we used separate scale factors for the main and satellite reflections. Upon doing so, the twin fractions converged to 0.50, within two times the standard uncertainty, indicating that the crystal has balanced twinning.

In the final model, all the atoms are refined anisotropically, and first order modulations were applied to the harmonic atomic displacement parameters for all atoms but Zn6, with its relatively short and discontinuous domains. In addition, the Δ*x*_4_ widths for Zn5 and Zn6 were refined with the restraint that the edges of their atomic domains are aligned for clean transitions in the physical cross-sections, as in Δ*x*_4_[Zn6] = ½ + ½σ_3_ + (1 − Δ*x*_4_[Zn5]), yielding values of 0.988 (3) and 0.664 (4), respectively. The refined model positions for the Zn5 and Zn6 sites are compared to the Fourier electron density contours in Figs. 4 ▸(*c*) and 4 ▸(*d*). In both cases, the model consists of slanted lines that align closely with the density features.

### 3.4. Structural interpretation of the modulated model

The nature of the modulation pattern is simple to interpret in terms of the disordered channels of the EuMg_5+*x*_-type form of YZn_5+*x*_ described previously [Fig. 1 ▸(*a*)] and the (3+1)D Fourier electron densities for the corresponding atomic sites in the current structure. The Y and Zn1–Zn4 sites define the host structure of the EuMg_5+*x*_ type, and their electron density features are well described by harmonic functions with relatively small amplitudes (see Fig. S2 in the supporting information). By contrast, the Zn5 and Zn6 sites corresponding to the occupants of the channels have discontinuous atomic domains, indicating much stronger modulations. The Zn5/Zn6 sites can thus be seen as the major drivers of the modulation, with the positional displacements of the remaining atoms serving as adjustments to accommodate the resulting changes in their local environments.

**Figure 5:**
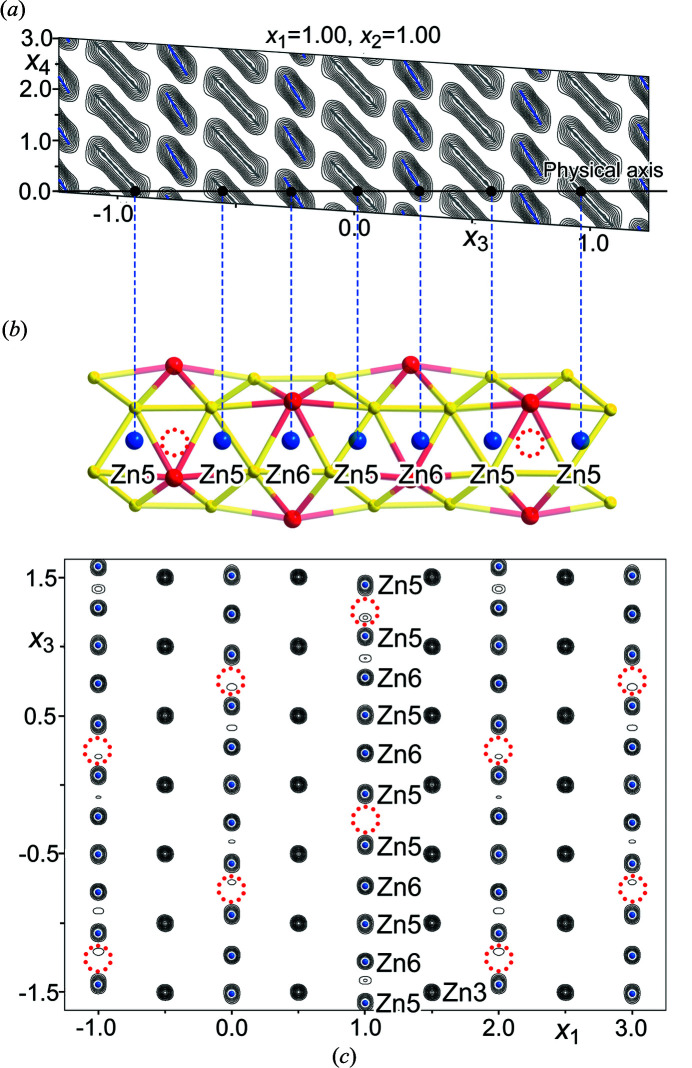
Connection between the (3+1)D Fourier electron density and structural features in physical space. (*a*) Fourier electron density contours corresponding to the Zn5 and Zn6 sites in an *x*_3_ *x*_4_ section. (*b*) The corresponding modulated channel containing Zn5 and Zn6 atoms in physical space. (*c*) Modulated Zn5 and Zn6 sites in physical space laid over the Fourier electron density map of the *xz* plane of a 3D density constructed from the (3+1)D model (*t*-section). Such sections can appear somewhat messy due to the density features being broader than the model’s atomic domains. The *x*_4_ offset of the cross-section off of the origin in (3+1)D space has been adjusted (*t* = 0.16) to reveal well separated density peaks along the channel. Vacant Zn6 positions are denoted by red dotted circles.

The structural consequences of the Zn5/Zn6 site modulations become clearer when looking at a larger cross-section for the (*x*_3_, *x*_4_) plane containing them (Fig. 5 ▸). Zn5 and Zn6 domains alternate as we move from left to right on the physical axis, corresponding to the centers of the flattened octahedra and tricapped trigonal prisms whose alternation builds up the channel walls. While the Zn5 and Zn6 sites are made distinct by their association with these two different polyhedra, their domains show a high degree of correlation: their electron density features are both slanted from upper left to lower right, and following one domain down this slope brings one to right to the tip of the next atomic domain, on the other side of a small gap.

In fact, if one were to shrink these gaps, the Zn5 and Zn6 domains would coalesce into diagonal stripes that run with a slant relative to the **a** *_s_*_4_ axis. Such a continuous slanted domain would encode a series of equally spaced Zn atoms whose spacing in physical space is mismatched with respect to the remainder of the structure (and determined by the angle of the slant). The high degree to which this pattern approximates a series of parallel diagonally oriented lines reveals that the Zn atoms in the channel have moved towards an equal spacing that is independent of the host lattice, as in a composite structure (Yamamoto, 1993 ▸; Rohrer *et al.*, 2000 ▸; Sun *et al.*, 2007 ▸). Indeed, the structure can be formally expressed as a composite structure in which the Zn6/Zn5 atoms occupying the channels are described as having their own lattice vector, **c**_guest_, which is incommensurate to that of the host structure, **c**_host_, with **c**_guest_ = **c**_host_/(3 + σ_3_). To perform refinements within this composite formalism, one first transforms the cell to a 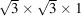 supercell (to obtain an axial **q** vector) and then assigns the reciprocal basis vectors of the guest composite part (the channel occupants) as **c**_guest_* = 3**c**_host_* + **q**_host_ and **q**_guest_ = 2**c**_host_*. This corresponds to the following transformation matrix connecting the *hklm* indices of each reflection in terms of the host and guest composite parts:

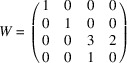

When implemented, this provides the channel occupants with their own axis in the (3+1)D model. However, two crenel functions are still needed, representing different scenarios for the tricapped trigonal prismatic and flattened octahedral holes in the channels, and the overall *R* factors are quite similar to those obtained above.

While a composite model would normally suggest that all points along the host cell’s channels can be populated by guest atoms, the gaps between the Zn5 and Zn6 domains suggest that certain positions are not suitable. In fact, the gaps correspond approximately in physical space to the centers of flattened Zn_3_Y_3_ octahedra that lie on opposite sides of the Zn6 sites. An explanation for these gaps can be discerned in the somewhat steeper slope of the Zn6 atomic domains than for the Zn5 atoms. The higher verticality here corresponds to a tighter localization around the average Zn6 position, indicating a preference for the center of the tricapped trigonal prism, where the Y–Zn contacts are shorter.

The features we have discussed so far explain the component of the modulation along the **c** axis of the EuMg_5+*x*_ type. The full **q** vector, **q** = 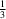 a* + 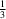 b* + 0.3041c*, also contains components along the perpendicular directions a* and b*, indicating that the occupation patterns of neighboring channels differ from each other. In the (3+1)D model, the channels are translationally related by steps of Δ*x*_1_ = ±1 and Δ*x*_2_ = ±1. In this construction, the non-zero components of σ_1_ and σ_2_ are reflected in the tilting of the **a** *_s_*_1_ and **a** *_s_*_2_ axes off the physical **a** and **b** axes, such that points along these axes are given by (*x*_1_, 0, 0, +σ_1_ *x*_1_ + *t*) and (0, *x_2_*, 0, +σ_2_ *x*_2_ + *t*), respectively, where *t* is the intercept with the ***a*** *_s_*_4_ axis chosen when making the 3D physical cross-section (and is arbitrary in the case of an incommensurate structure). Since σ_1_ and σ_2_ are both equal to 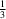 [as required by the superspace group 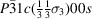 ], moving from one channel to one of its neighbors along **a**_basic_ or **b**_basic_ results in a shift of the modulation phase by 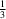 . These shifts are evident in the electron density maps for the 3D cross-section [*t*-maps, Fig. 5 ▸(*c*)], where the Zn6 vacancies in neighboring channels are offset from each other by steps of 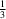 of the spacing between the vacancies along the channel.

**Figure 6:**
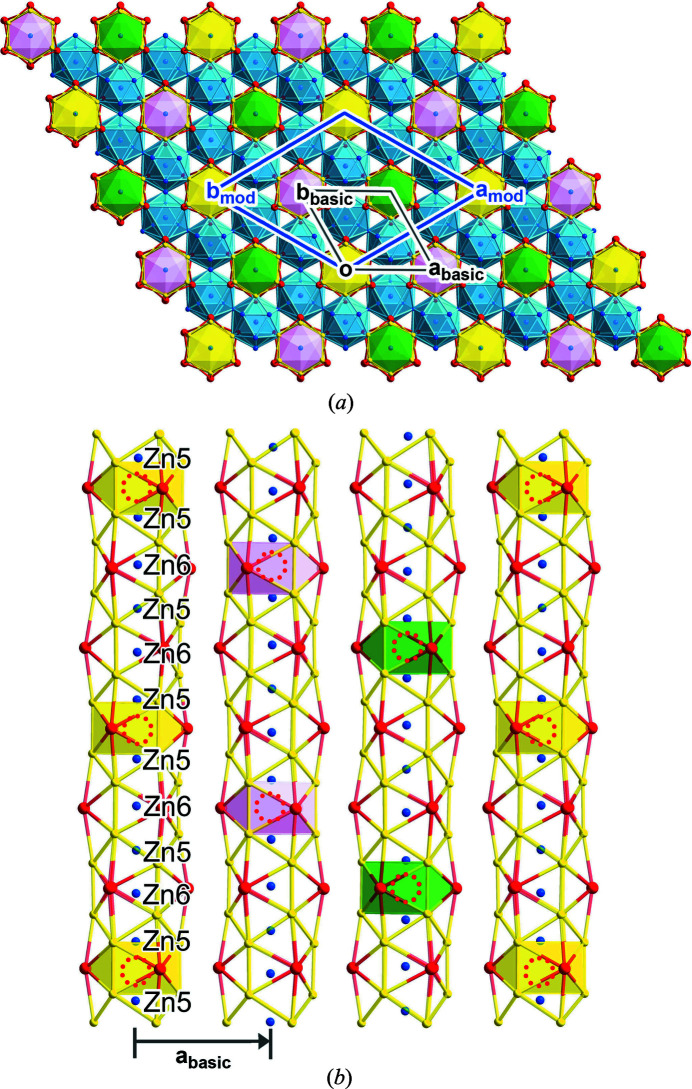
Incommensurately modulated YZn_5+*x*_ (*x* = 0.217) structure. (*a*) Overview of the structure viewed down [001]. (*b*) The modulated Zn channels with the one-third shift pattern (yellow, pink, and green). Vacant Zn6 positions are denoted by red dotted circles.

This picture can be compared to the 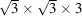 superstructure predicted previously from tracing paths of chemical pressure between channels through the tcp units. In this model, every third Zn6 site is vacant within each channel, creating a threefold superstructure along **c**. The occupation patterns of neighboring channels are connected through rhombohedral centering, with the primitive cell vectors being **a**_prim_ = **a**_basic_ + **c**_basic_, **b**_prim_ = **b**_basic_ + **c**_basic_, and **c**_prim_ = −**a**_basic_ − **b**_basic_ + **c**_basic_. Transforming this arrangement to the hexagonal setting gives rise to the 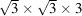 supercell. In Fig. 6 ▸, we plot the 3D structure of the incommensurately modulated form of YZn_5+*x*_ generated from our (3+1)D model. First in Fig. 6 ▸(*a*), we show a view down *c*, color-coding the different channels according to the relative heights of the channel occupation patterns. In this case, the **a** and **b** vectors of the 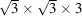 supercell represent true crystallographic translations, labeled **a**_mod_ and **b**_mod_, due to the commensurability of the modulation along these directions. This corresponds to the predicted pattern in the *ab* plane, as can be seen by comparison with Fig. 1 ▸(*b*).

Next, in Fig. 6 ▸(*b*), we examine the occupation patterns of neighboring channels in more detail. In each channel, every third Zn6 atom is missing in this portion of the structure (red dotted circles), but as these positions are not translationally equivalent points in the basic structure (the EuMg_5+*x*_-type unit cell has two Zn6 sites per cell along *c*) the overall pattern repeats every six Zn6 positions. The neighboring channels are shifted out of step by one Zn6 site, with the Zn6 coordination environments in the same orientation becoming shifted by 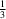 of the approximate supercell vector along *c*. If the structure were to be commensurate, this would correspond in all respects to the predicted model.

The close approximation of σ_3_ to 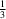 raises the question of whether a commensurate model may be warranted. To answer this question, we attempted refinements in which σ_3_ was set to 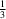 and the structure was treated as commensurate in terms of the structure factor calculations. Regardless of which *t*_0_ value was chosen, however, the *R* factors for the model were unreasonably high [overall *R* (*I* > 3σ) > 10.0 versus 2.7 for the incommensurately modulated model]. The incommensurability of the phase is further supported by the observation that the σ_3_ value obtained from the diffraction patterns of different crystals can vary from 0.304 to 0.331 (in fact, some slight splitting of the satellites into closely spaced pairs of σ_3_ values can be detected in the lattice reconstructions). As such, the spacing of Zn atoms along the structure’s channels appears not to be locked into registry with the *c* parameter of the host structure.

How, then, does the incommensurately modulated structure differ from the idealized threefold superstructure along *c*? As we mentioned above, the σ_3_ value in the (3+1)D model controls the average spacing of the Zn atoms occupying the channels, **c**_guest_, relative to the *c* parameter of the host sublattice’s average structure, **c**_host_, as in **c**_guest_ = **c**_host_/(3+σ_3_). For σ_3_ = 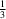 , this gives rise to a commensurate arrangement with three **c**_host_ = ten **c**_guest_, *i.e.* ten Zn5/Zn6 atoms in a threefold supercell of the disordered EuMg_5+*x*_-type structure, matching that of the originally predicted superstructure with composition YZn_5.225_. For lower σ_3_ values, the average spacing of the channel atoms increases, leading to a lower overall Zn content of 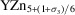 , *e.g.* YZn_5.217_ for σ_3_ = 0.3041, with each atom in the channel locking into a nearby Zn5 or Zn6 atomic domain.

## 4. Conclusions

In our earlier analysis of EuMg_5+*x*_-type YZn_5+*x*_, we identified paths of chemical pressures between its disordered channels that point to a potential ordered superstructure. In this article, we have confirmed this picture experimentally, demonstrating that, through a change to the synthetic procedure, YZn_5+*x*_ crystals with satellite reflections from channel order can be obtained. In this pattern, approximately every third Zn6 position within the channel is vacant, with the remaining occupants exhibiting displacements that serve to smooth out the spacing between them. Between neighboring channels, shifts in the pattern arise to stagger the positions of the vacancies, which we had predicted would minimize the accumulation of packing tensions along paths of inter-channel contacts. Rather than the expected 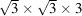 supercell of the EuMg_5+*x*_ type, however, the pattern is incommensurate along the **c** axis, as revealed by variability in the σ_3_ parameter of the **q** vector and the satellite intensity distributions.

These results contribute to a growing theme in the structural chemistry of intermetallics: the communication of information between different regions of a crystal structure through paths of chemical pressures (CPs). For example, the remarkably large hysteresis in the cubic to tetragonal phase transition of PtGa_2_ was interpreted in terms of the tight coordination of atomic motion imposed by its CP scheme (Mitchell Warden *et al.*, 2018 ▸). Similarly, the unusual, slanted domains of the trimer–monomer patterns in the incommensurately modulated IrSi structure (Mitchell Warden & Fredrickson, 2019 ▸), as well as the form of the low-temperature superstructure and high-temperature incommensurability in PdBi (Folkers *et al.*, 2020 ▸), were traced to the domino-like propagation of atomic displacements that create emergent, long-range patterns. In these cases, the explanations for the detailed choreographies of atomic motions benefited from knowing the structural solution beforehand. With the example of YZn_5+*x*_, we see more clearly how this approach can be used in a predictive fashion: beginning with CP schemes for a handful of ordered models, the Zn site occupancies in the average structure, and hints from the literature about the size of the expected supercell, an ordering pattern could be proposed, which could then be successfully realized synthetically.

An open challenge highlighted by this work is the question of whether such ordering patterns will adopt commensurate or incommensurate arrangements. Could the incommensurability in YZn_5+*x*_ have been predicted ahead of time? In this case, the CP features offer clear indications of how the occupation patterns of neighboring channels are coupled, but give less guidance on the preferred sequence along the channels. Instead, our original model used experimental Zn occupancies in the average structure, and assumed the vacancies would be distributed at regular intervals. There is room within that scheme for these intervals to be mismatched from the **c** period of the host structure, but there was not an obvious reason for this being advantageous. One potential hint at a driving force for more Zn6 occupancy is seen in the large positive CPs taken on by Zn5 sites neighboring a Zn6 vacancy, as they move in to smooth out the Zn–Zn distances along the channel. Incommensurability would allow the structure to tune the Zn6 vacancy concentration to balance the benefits of a sparser occupation of the channels with the energetic costs to the Zn5 sites neighboring the vacancies. It will be interesting to see whether theoretical models, based on CP analysis or other tools, can be developed to detect such situations where incommensurability provides an opportunity to balance competing factors.

## Supplementary Material

Crystal structure: contains datablock(s) I. DOI: 10.1107/S2052520623005292/dk5117sup1.cif

Structure factors: contains datablock(s) I. DOI: 10.1107/S2052520623005292/dk5117Isup2.hkl

Sections S1, S2 (Table S1), S3 (Table S2), S4 (Fig. S1, S2, S3). DOI: 10.1107/S2052520623005292/dk5117sup3.pdf

uGPEM0uGBx9

CCDC reference: 2269662

## Figures and Tables

**Table 1:** Crystallographic data for the modulated form YZn_5+*x*_

Chemical formula	YZn_5.217_
Wavelength dispersive X-ray spectroscopy composition	YZn_5.26 (3)_
*a* (Å), *c* (Å)	8.8811 (1), 9.2057(1)
Volume (basic cell, Å^3^)	628.812 (12)
Z	6
Modulation wavevector†	**q** = 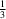 a* + 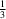 b* + 0.3041c*
Superspace group‡	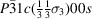
Crystal dimensions (mm)	0.193 × 0.137 × 0.092
Crystal color, habit	Metallic gray, prismatic
Data collection temperature	Ambient
Radiation source, λ (Å)	Mo *K*α sealed tube, 0.71073
Absorption correction	Analytical
Min, max transmission	0.024, 0.125
θ_min_, θ_max_	3.27, 27.87
	
Refinement method	*F* ^2^
No. of reflections	24910
No. of unique reflections [*I* > 3σ(*I*), all]	777, 1450
*R*_int_ [*I* > 3σ(*I*), all]	5.24, 5.55
No. of parameters, constraints	90, 0
	
Overall reflections	
*R*[*I* > 3σ(*I*)], *R*_w_[*I* > 3σ(*I*)]	2.72, 7.88
*R*(all), *R*_w_(all)	5.60, 8.76
*S*[*I* > 3σ(*I*)], *S*(all)	1.94, 1.53
	
Main reflections	
*R*[*I* > 3σ(*I*)], *R*_w_[*I* > 3σ(*I*)]	1.76, 6.58
*R*(all), *R*_w_(all)	1.90, 6.63
	
Satellite reflections	
*R*[*I* > 3σ(*I*)], *R*_w_[*I* > 3σ(I)]	11.12, 18.87
*R*(all), *R*_w_(all)	24.85, 23.72
Δρ_max_, Δρ_min_(e Å^−3^)	4.73, −4.90

† Refined **q** vector without symmetry constraints: **q** = 0.3337(6) a* + 0.3314(6) b* + 0.3041(7) c*.

‡ Symmetry operations: (*x*_1_, *x*_2_, *x*_3_, *x*_4_), (−*x*_2_, *x*_1_ − *x*_2_, *x*_3_, −*x*_2_ + *x*_4_), (−*x*_1_ + *x*_2_, −*x*_1_, *x*_3_, −*x*_1_ + *x*_4_), (−*x*_2_, −*x*_1_, −*x*_3_ + ½, −*x*_4_ + ½), (−*x*_1_ + *x*_2_, *x*_2_, −*x*_3_ + ½, *x*_2_ − *x*_4_ + ½), (*x*_1_, *x*_1_ − *x*_2_, −*x*_3_ + ½, *x*_1_ − *x*_4_ + ½), (*x*_2_, −*x*_1_ + *x*_2_, −*x*_3_, *x*_2_ − *x*_4_), (*x*_1_ − *x*_2_, *x*_1_, −*x*_3_, *x*_1_ − *x*_4_), (*x*_2_, *x*_1_, *x*_3_ + ½, *x*_4_ + ½), (*x*_1_ − *x*_2_, − *x*_2_, *x*_3_ + ½, −*x*_2_ + *x*_4_ + ½), (−*x*_1_, −*x*_1_ + *x*_2_, *x*_3_ + ½, −*x*_1_ + *x*_4_ + ½), (−*x*_1_, −*x*_2_, −*x*_3_, −*x*_4_)

**Table 2:** Refined atomic coordinates for the basic structure of YZn_5+*x*_

Site	Multiplicity	*x*	*y*	*z*	*U*_equiv_	Occupancy
Y	6	0.61475 (10)	0.80738 (5)	¼	0.0107 (3)	1
Zn1	6	0.86508 (10)	0.43254 (5)	¼	0.0117 (3)	1
Zn2	4	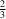	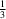	0.51174 (10)	0.0122 (3)	1
Zn3	6	1	½	½	0.0123 (6)	1
Zn4	12	0.8383 (2)	0.67858 (8)	0.41030 (6)	0.0139 (5)	1
Zn5	2	1	1	0	0.0196 (12)	0.988 (4)
Zn6	2	1	1	¼	0.0140 (9)	0.664 (4)

**Table 3:** Refined atomic displacement parameters for the basic structure of YZn_5+*x*_

Site	*U*_11_	*U*_22_	*U*_33_	*U*_12_	*U*_13_	*U*_23_
Y	0.0123 (4)	0.0104 (3)	0.0102 (3)	0.00617 (19)	0	0.0067 (7)
Zn1	0.0118 (4)	0.0130 (3)	0.0099 (4)	0.0059 (2)	0	0.0002 (12)
Zn2	0.0115 (3)	0.0115 (3)	0.0134 (5)	0.00577 (16)	0	0
Zn3	0.0115 (4)	0.0122 (9)	0.0108 (4)	0.0040 (9)	−0.0017 (3)	−0.0004 (11)
Zn4	0.0162 (6)	0.0147 (4)	0.0140 (3)	0.0102 (7)	0.0002 (8)	0.0006 (2)
Zn5	0.0149 (6)	0.0149 (6)	0.029 (3)	0.0074 (3)	0	0
Zn6	0.0144 (7)	0.0144 (7)	0.013 (2)	0.0072 (4)	0	0
