# Supplementary material for: As predicted and more: modulated channel occupation in YZn5+x
Source: Acta Crystallogr B Struct Sci Cryst Eng Mater. 2023 Jul 7;79(Pt 4):320–9. doi: 10.1107/S2052520623005292 (PMC10410308; doi:10.1107/S2052520623005292)
Supplement: Supplementary file 3 [file b-79-00320-sup3.pdf]

STRUCTURAL SCIENCE
CRYSTAL ENGINEERING
MATERIALS

Volume 79 (2023)

Supporting information for article:

As predicted and more: modulated channel occupation in YZn5+*x*

Rie T. Fredrickson and Daniel C. Fredrickson

S1. Derivations of Relationships for the (3+1)D Modeling of YZn_{5+x}

Crenel function Δx_4 parameters for the Zn5 and Zn6 sites. The Δx_4 parameter for an atomic domain described with a crenel function gives the length of the interval along the x_4 (centered at $x_{4,o}$) for which the atom is present. With the slanting of the atomic domains in the (x_3,x_4) planes in Figure 5, the upper left tip of the Zn6 domains closely approaches the lower right tip of the Zn5 domain whose center is displaced by (-¼,¾) from the Zn6 center. On the other side of the Zn6 domain, its lower right tip comes close to the upper left tip of the Zn5 domain whose center is offset from the Zn6 center by (¼,-¾). A continuous coverage for the x_4 axis by each Zn5/Zn6 line can be obtained by lining up the ends of the domains so that their tips occur at the same lines perpendicular to the x_4 axis, parallel to the physical z axis. On the left side of the Zn6 domain, this will occur when ¾-Δx_4[Zn5]/2+σ_3/4 = Δx_4[Zn6]/2, where σ_3/4 is the shift along x_4 needed to stay on the physical z-axis after moving ¼ along x_3. Similarly, on the right side the match along z is achieved with -Δx_4[Zn6]/2 + σ_3/4 = -¾ + Δx_4[Zn5]/2. Using either equation, we obtain Δx_4[Zn6] = ½ + σ_3/2 + (1 - Δx_4[Zn5]). In the special case where Δx_4[Zn5] = 1, this yields Δx_4[Zn6] = ½ + σ_3/2.

Relationship between the $\mathbf{c}_{host}$ and $\mathbf{c}_{guest}$ in the composite description of YZn_{5+x}. From Figure 5, note that the slanted lines created by the Zn5 and Zn6 atomic domains have an average slope of $\Delta x_4/\Delta x_3$ = -3/1. If we label these slanted lines with the index j, the coordinates on the points of line j will be given by ($x_{3,j}$,0) + ε(-1,3), where $x_{3,j}$ is the intercept of the line with the x_3 axis, and ε is a free variable. The z coordinate in physical space of the atom arising from line j can be derived by setting $\mathbf{z}$ = (x_3, x_4) = (x_3, σ_3 x_3) equal to ($x_{3,j}$,0) + ε(-1,3). From this we find $x_3 = x_{3,j} - \varepsilon$ and $\sigma_3 x_3 = 3\varepsilon$. Solving for x_3 in terms of $x_{3,j}$, we arrive at $x_3 = x_{3,j}/(1+\sigma_3/3)$ as the coordinate where the line intersects the physical z axis. As the slanted lines divide each repeat vector along x_3 into three intervals, the average space between two neighboring Zn atoms along z in the channel is one third of $1/(1+\sigma_3/3)$, i.e. $1/(3+\sigma_3)$. Since these numbers represent fractions of the c lattice vector of the host average structure, we have $\mathbf{c}_{guest}$ = $\mathbf{c}_{host}/(3+\sigma_3)$.

The composition of YZn_{5+x} in terms of σ_3. One average cell of the host structure has the contents Y_6Zn_{28} and contains one channel. The channel contains Zn atoms spaced at intervals of $\mathbf{c}_{guest}$, giving on average c_{host}/c_{guest} = $3+\sigma_3$ atoms per host cell. Altogether, the composition of the phase is then $Y_6Zn_{28+3+\sigma 3}$ = $Y_6Zn_{31+\sigma 3}$ or $YZn_{5+(1+\sigma 3)/6}$.

S2. Crystallographic Information Tables for the (3+1)D model of YZn_{5+x}

Detailed tables are given describing the atomic coordinates, atomic displacement parameters, and modulation functions refined for the (3+1)D model of YZn_{5+x}.

Table S1 Modulation parameters for YZn_{5+x}.

Crenel Functions	Atomic sites	Δ	x_{40}
	Zn5	0.988(4)	0.5
	Zn6	0.664(4)	0.75

Legendre Polynomial (xyz)	Atomic sites	N	$S_{x,n}$(odd)	$S_{y,n}$(odd)	$S_{z,n}$(odd)	$S_{x,n}$(even)	$S_{y,n}$(even)	$S_{z,n}$(even)
	Zn5	1	0	0	-0.0996(9)	0	0	0
	Zn6	1	0	0	-0.0447(8)	0	0	0

Harmonic (xyz)	Atomic sites	N	$U_{x,n}$(sin)	$U_{y,n}$(sin)	$U_{z,n}$(sin)	$U_{x,n}$(cos)	$U_{y,n}$(cos)	$U_{z,n}$(cos)
	Y	1	0.00386(10)	0.0044(2)	-0.00165(19)	0.0102(3)	0.00417(17)	0.00062(7)
	Zn1	1	-0.0021(3)	0.00028(17)	0.00036(10)	-0.00096(12)	-0.0035(5)	-0.0008(2)
	Zn2	1	-0.0014(2)	0.0035(3)	0	-0.0048(3)	-0.0036(3)	0
	Zn3	1	-0.0039(3)	-0.0020(3)	0.0014(2)	0	0	0
	Zn4	1	-0.0035(2)	-0.0043(2)	0.00202(19)	-0.0039(2)	-0.0094(2)	-0.00357(19)

Harmonic (ADP)	Atom sites	N	$U_{11,n}$(sin)	$U_{22,n}$(sin)	$U_{33,n}$(sin)	$U_{12,n}$(sin)	$U_{13,n}$(sin)	$U_{23,n}$(sin)
	Y	1	0.0014(4)	-0.0005(7)	0.0002(4)	-0.0002(7)	-0.0004(8)	-0.0007(5)
	Zn1	1	-0.0002(12)	-0.0011(9)	0.0003(12)	-0.0010(7)	0.0001(4)	0.0004(8)
	Zn2	1	-0.0007(9)	0.0013(9)	0	0.0006(13)	0.0004(11)	0.0009(9)
	Zn3	1	0	0	0	0	0	0
	Zn4	1	0.0001(10)	0.0035(10)	-0.0039(10)	0.0015(8)	0.0001(8)	0.0014(7)

Harmonic (ADP)	Atom sites	N	$U_{11,n}$(cos)	$U_{22,n}$(cos)	$U_{33,n}$(cos)	$U_{12,n}$(cos)	$U_{13,n}$(cos)	$U_{23,n}$(cos)
	Y	1	0.0036(9)	0.001(3)	0.0006(10)	0.0022(6)	0.0001(3)	-0.0013(18)
	Zn1	1	-0.0001(5)	0.002(2)	0.0002(5)	0.002(2)	-0.0002(9)	0.0000(7)
	Zn2	1	0.0011(12)	0.0001(11)	0	0.0011(7)	-0.0008(12)	0.0001(14)
	Zn3	1	0.0025(13)	0.0004(13)	-0.0042(12)	0.0016(11)	0.0000(12)	-0.0008(11)
	Zn4	1	-0.0019(10)	-0.0017(9)	0.0005(9)	-0.0012(8)	-0.0003(8)	-0.0012(7)

Legendre Polynomial (ADP)	Atomic sites	N	$S_{11,n}$(odd)	$S_{22,n}$(odd)	$S_{33,n}$(odd)	$S_{12,n}$(odd)	$S_{13,n}$(odd)	$S_{23,n}$(odd)
	Zn5	1	0	0	0	0	0	0

Legendre Polynomial (ADP)	Atomic sites	N	$S_{11,n}$(even)	$S_{22,n}$(even)	$S_{33,n}$(even)	$S_{12,n}$(even)	$S_{13,n}$(even)	$S_{23,n}$(even)
	Zn5	1	-0.008(3)	-0.008(3)	-0.016(6)	-0.0038(13)	0	0

S3. Wavelength Dispersive X-ray Spectroscopy (WDS) Results

Table S2 WDS composition for different points and grains in the sample corresponding to YZn_{5+x}.

SAMPLE	LINE No.	Y AT%	Zn AT%	TOTAL	Zn/Y
pt1_grain1_brightBSE	85	16.1604	83.8396	100	5.187966
pt2_grain1_brightBSE	86	15.8964	84.1037	100	5.290739
pt3_grain1_brightBSE	87	15.6964	84.3036	100	5.370888
pt4_grain1_brightBSE	88	15.5378	84.4622	100	5.435918
pt5_grain1_brightBSE	89	15.4998	84.5002	100	5.451696
pt6_grain1_brightBSE	90	15.9108	84.0892	100	5.285039
pt7_grain1_brightBSE	91	15.8043	84.1957	100	5.327392
pt8_grain1_brightBSE	92	15.4566	84.5434	100	5.469728
pt9_grain2_brightBSE	101	15.5527	84.4473	100	5.429752
pt10_grain2_brightBSE	102	15.6593	84.3407	100	5.385981
pt11_grain2_brightBSE	103	15.6538	84.3462	100	5.388225
pt12_grain2_brightBSE	104	15.1953	84.8047	100	5.580982
pt13_grain2_brightBSE	105	15.4028	84.5972	100	5.492326
pt14_grain2_brightBSE	106	15.2985	84.7015	100	5.536589
pt15_grain2_brightBSE	107	15.3944	84.6056	100	5.495869
pt16_grain2_brightBSE	108	15.5266	84.4734	100	5.44056
pt17_grain2_brightBSE	109	15.3552	84.6448	100	5.512452
pt18_grain2_brightBSE	110	15.1264	84.8736	100	5.610958
pt19_grain3_brightBSE	116	16.1158	83.8842	100	5.205091
pt20_grain3_brightBSE	117	15.8624	84.1376	100	5.304216
pt21_grain3_brightBSE	118	16.2426	83.7574	100	5.15665
pt22_grain3_brightBSE	119	16.1863	83.8137	100	5.178064
pt23_grain3_brightBSE	120	15.9442	84.0558	100	5.271873
pt24_grain3_brightBSE	121	16.5746	83.4254	100	5.033328
pt25_grain3_brightBSE	122	16.152	83.848	100	5.191184
pt26_grain3_brightBSE	123	16.0078	83.9922	100	5.246955
pt27_grain3_brightBSE	124	16.2611	83.7389	100	5.149645
pt28_grain3_brightBSE	125	16.5746	83.4254	100	5.033328
pt29_grain4_brightBSE	136	16.2954	83.7047	100	5.136707
pt30_grain4_brightBSE	137	16.2054	83.7946	100	5.170783
pt31_grain4_brightBSE	138	16.5179	83.4821	100	5.054038
pt32_grain4_brightBSE	139	16.5504	83.4496	100	5.04215
pt33_grain4_brightBSE	140	16.337	83.663	100	5.121075
pt34_grain4_brightBSE	141	16.0835	83.9165	100	5.217552
pt35_grain4_brightBSE	142	16.2541	83.7459	100	5.152294
pt36_grain4_brightBSE	143	15.8327	84.1673	100	5.316042

pt37_grain4_brightBSE	144	16.2074	83.7926	100	5.170021
pt38_grain4_brightBSE	145	16.3884	83.6117	100	5.101883
pt39_grain4_brightBSE	156	16.5491	83.4509	100	5.042625
pt40_grain4_brightBSE	157	16.6303	83.3698	100	5.013127
pt41_grain4_brightBSE	158	16.1005	83.8995	100	5.210987
pt42_grain4_brightBSE	159	16.6497	83.3503	100	5.006114
pt43_grain4_brightBSE	160	16.4717	83.5284	100	5.071025
Average of 43 points		15.97959	84.02042		5.262554
Standard deviation		0.435342	0.435332		0.171816

S4. Plots of crystallographic information for YZn_{5+x}

Figure S1 Interatomic distances of incommensurately modulated YZn_{5+x}

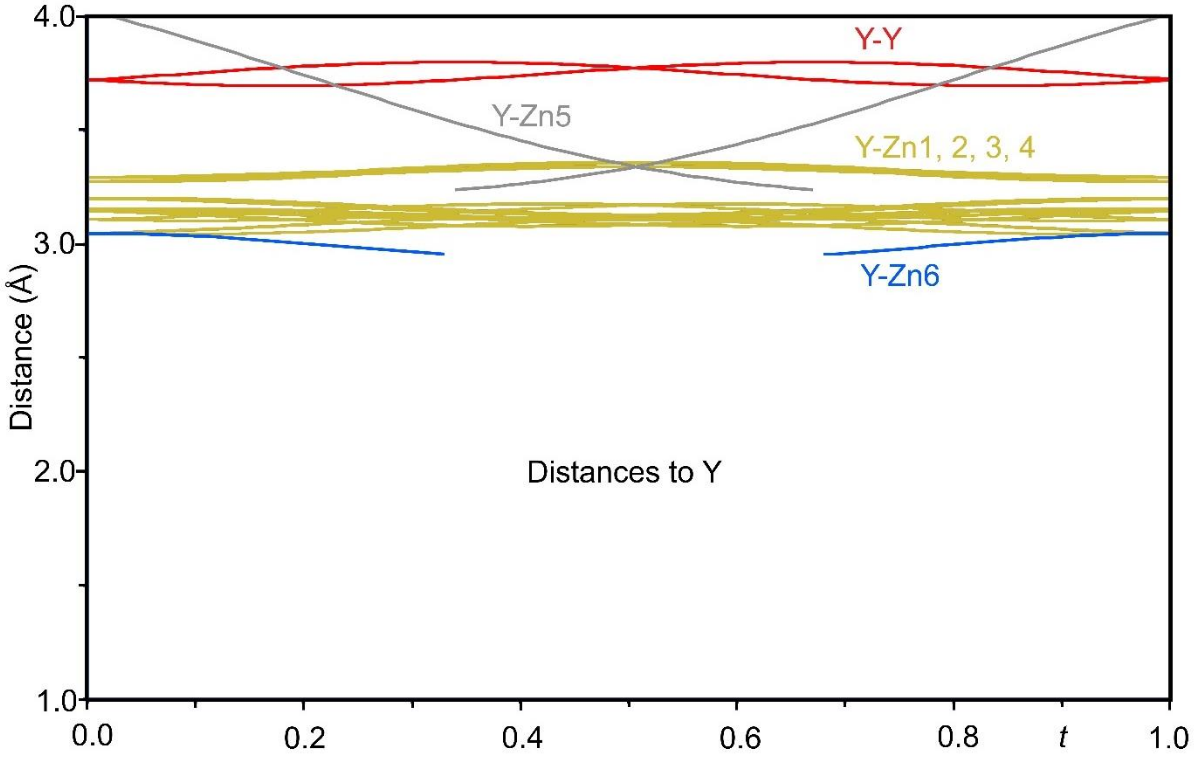

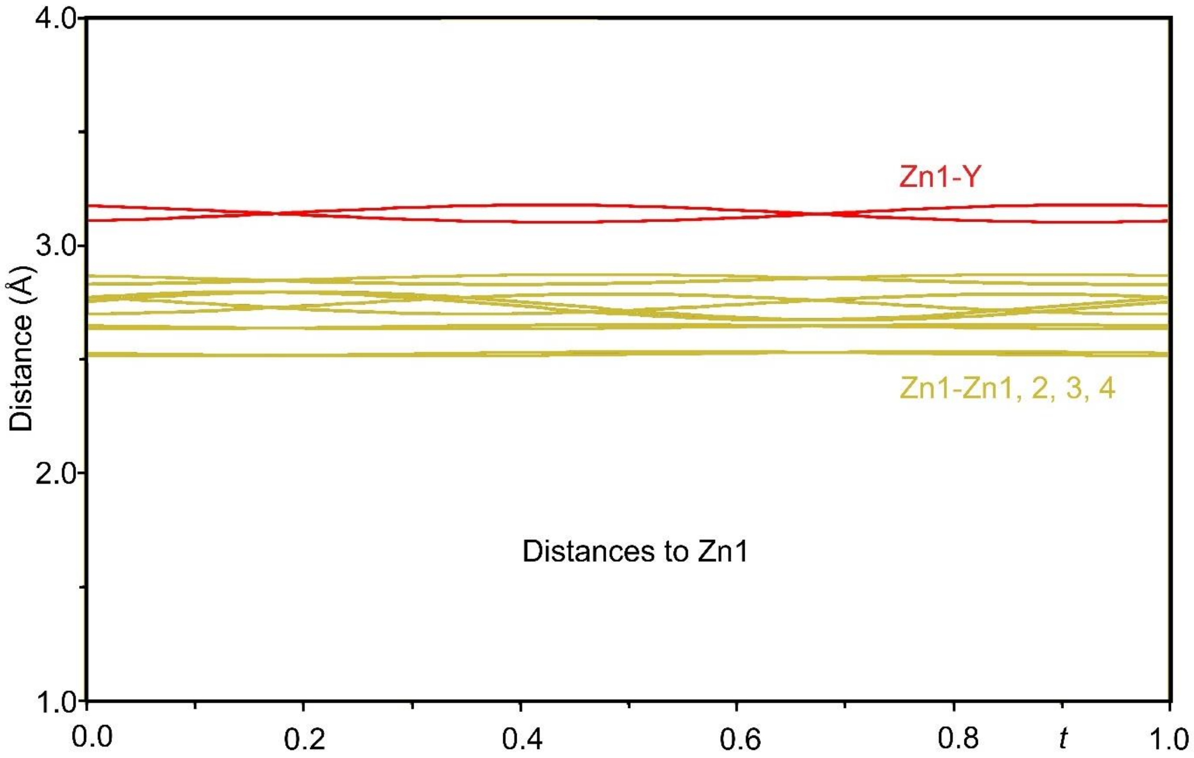
4.0
3.0
2.0
1.0
Distance (Å)
Zn1-Y
Zn1-Zn1, 2, 3, 4
Distances to Zn1
0.0
0.2
0.4
0.6
0.8
t
1.0

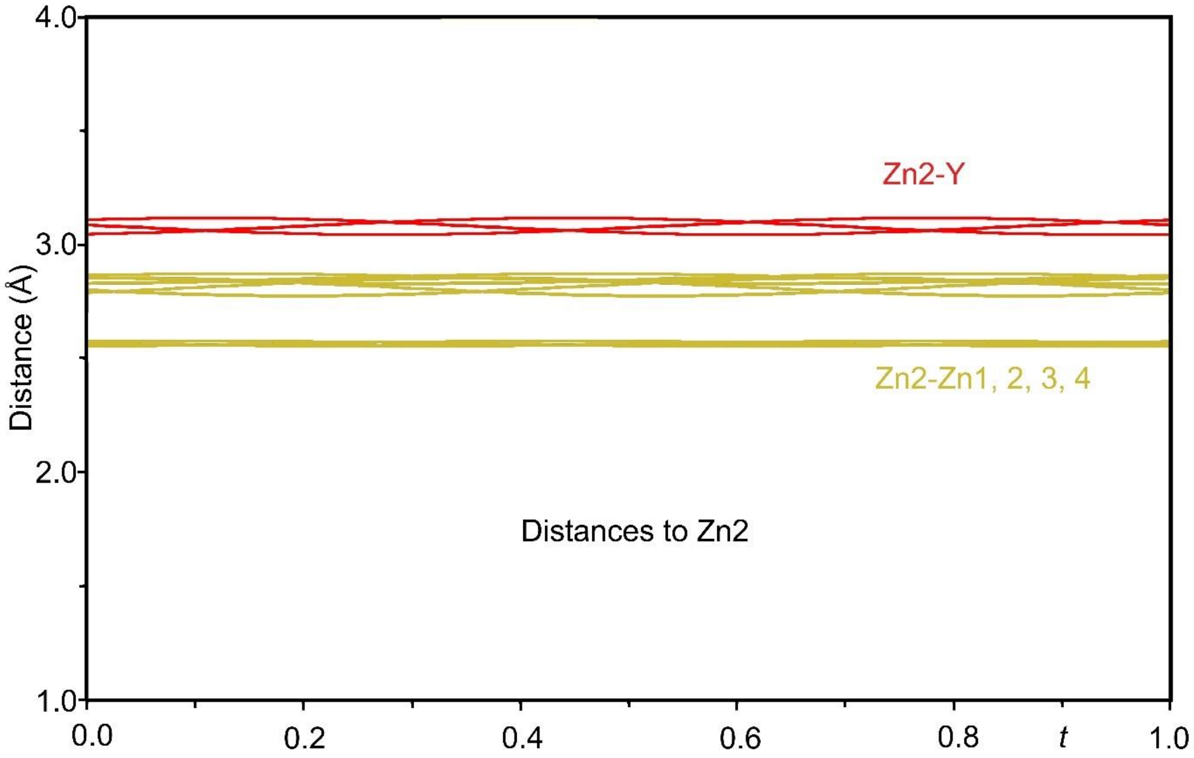
4.0
3.0
2.0
1.0
Distance (Å)
Zn2-Y
Zn2-Zn1, 2, 3, 4
Distances to Zn2
0.0
0.2
0.4
0.6
0.8
t
1.0

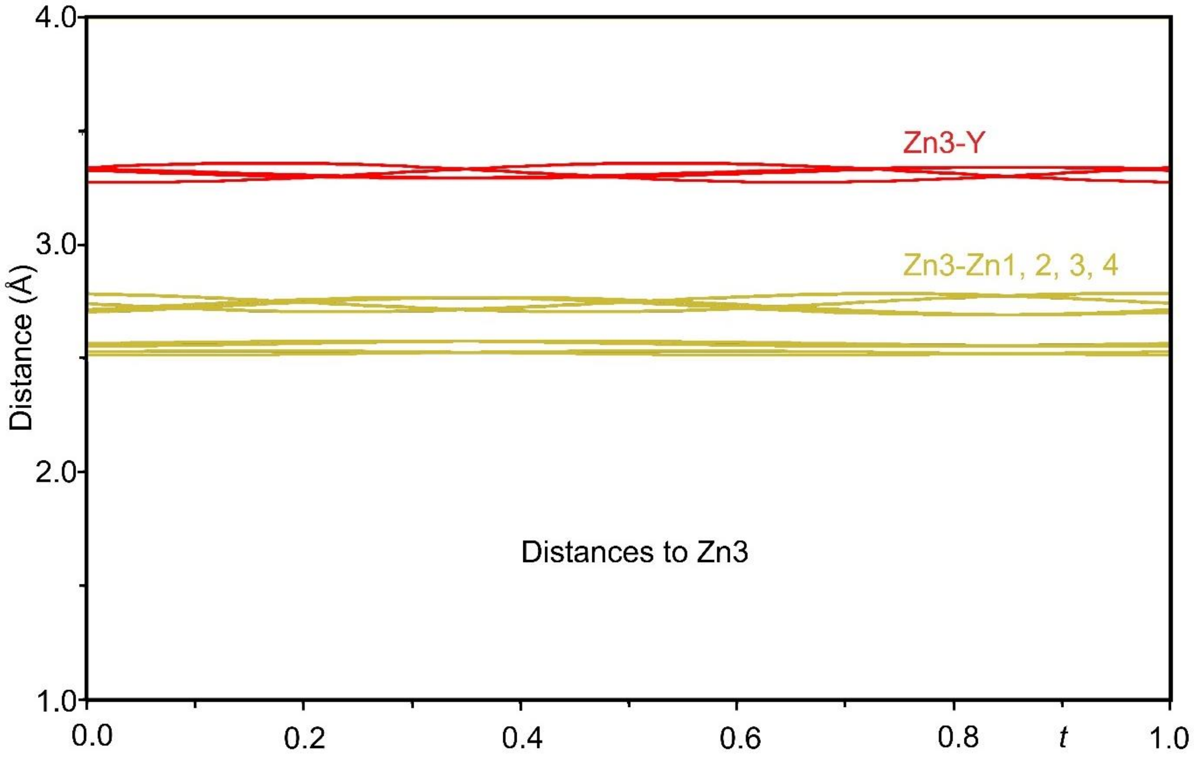
Zn3-Y
Zn3-Zn1, 2, 3, 4
Distances to Zn3
Distance (Å)
4.0
3.0
2.0
1.0
0.0
0.2
0.4
0.6
0.8
t
1.0

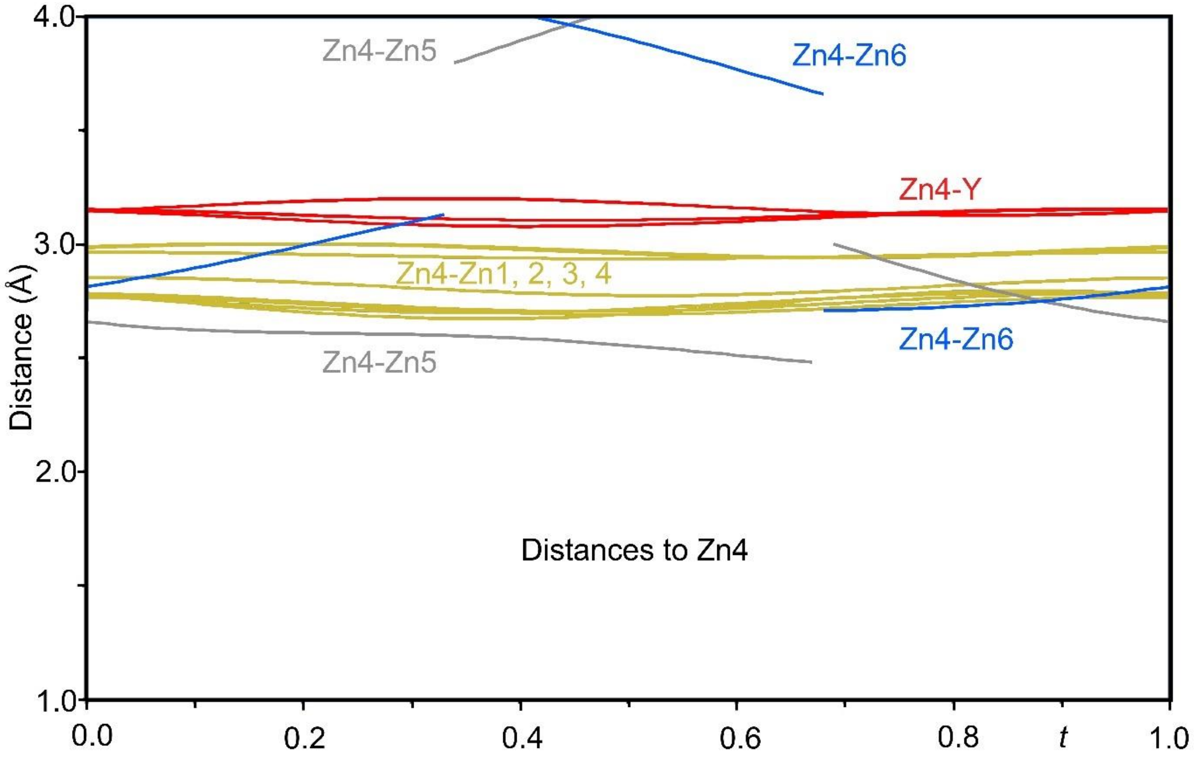
Zn4-Zn5
Zn4-Zn6
Zn4-Y
Zn4-Zn1, 2, 3, 4
Zn4-Zn6
Zn4-Zn5
Distances to Zn4
Distance (Å)
4.0
3.0
2.0
1.0
0.0
0.2
0.4
0.6
0.8
t
1.0

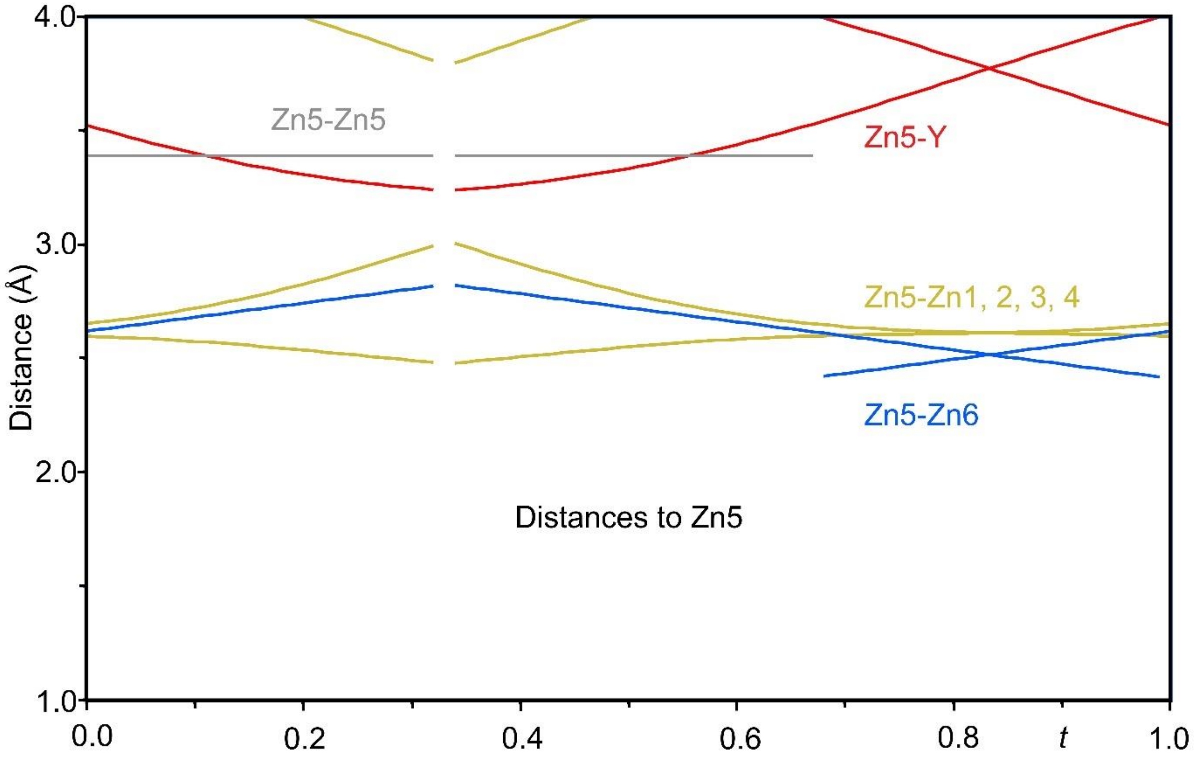
Zn5-Zn5
Zn5-Y
Zn5-Zn1, 2, 3, 4
Zn5-Zn6
Distances to Zn5
Distance (Å)
4.0
3.0
2.0
1.0
0.0
0.2
0.4
0.6
0.8
t
1.0

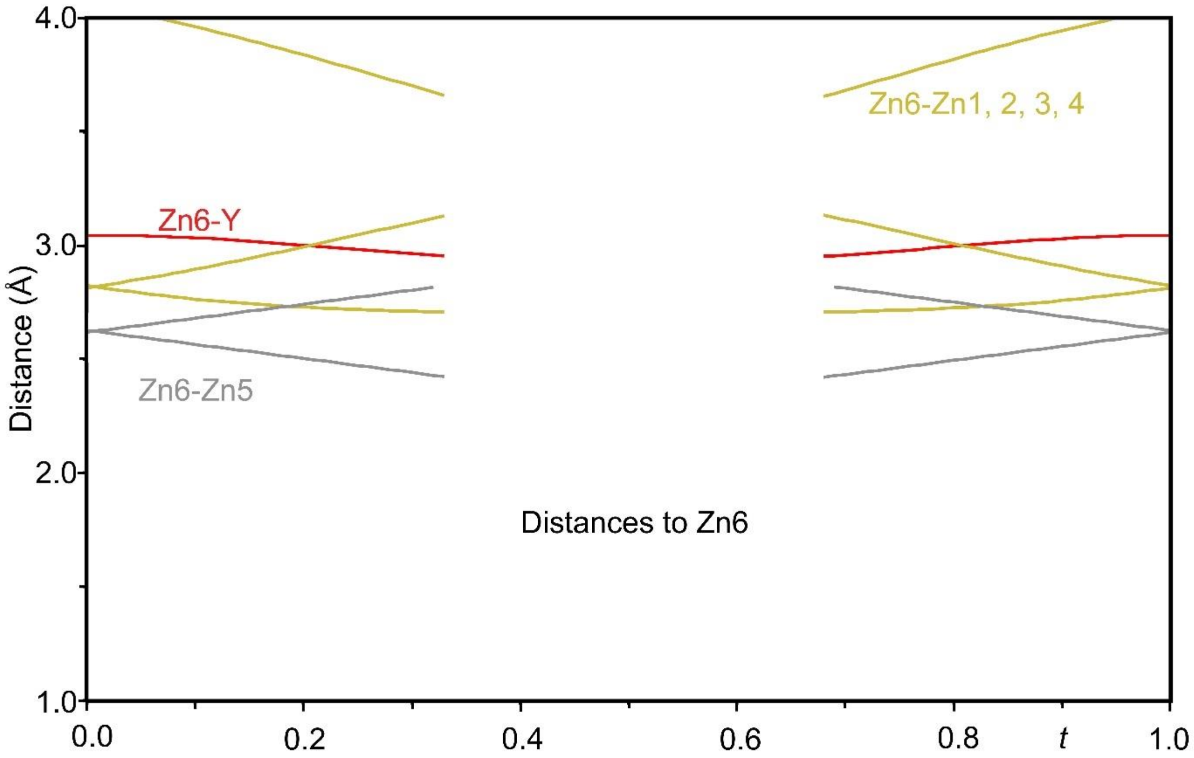
Zn6-Zn1, 2, 3, 4
Zn6-Y
Zn6-Zn5
Distances to Zn6
Distance (Å)
4.0
3.0
2.0
1.0
0.0
0.2
0.4
0.6
0.8
t
1.0

Figure S2 Observed Fourier electron density map cross sections centered on atomic positions in YZn_{5+x}.

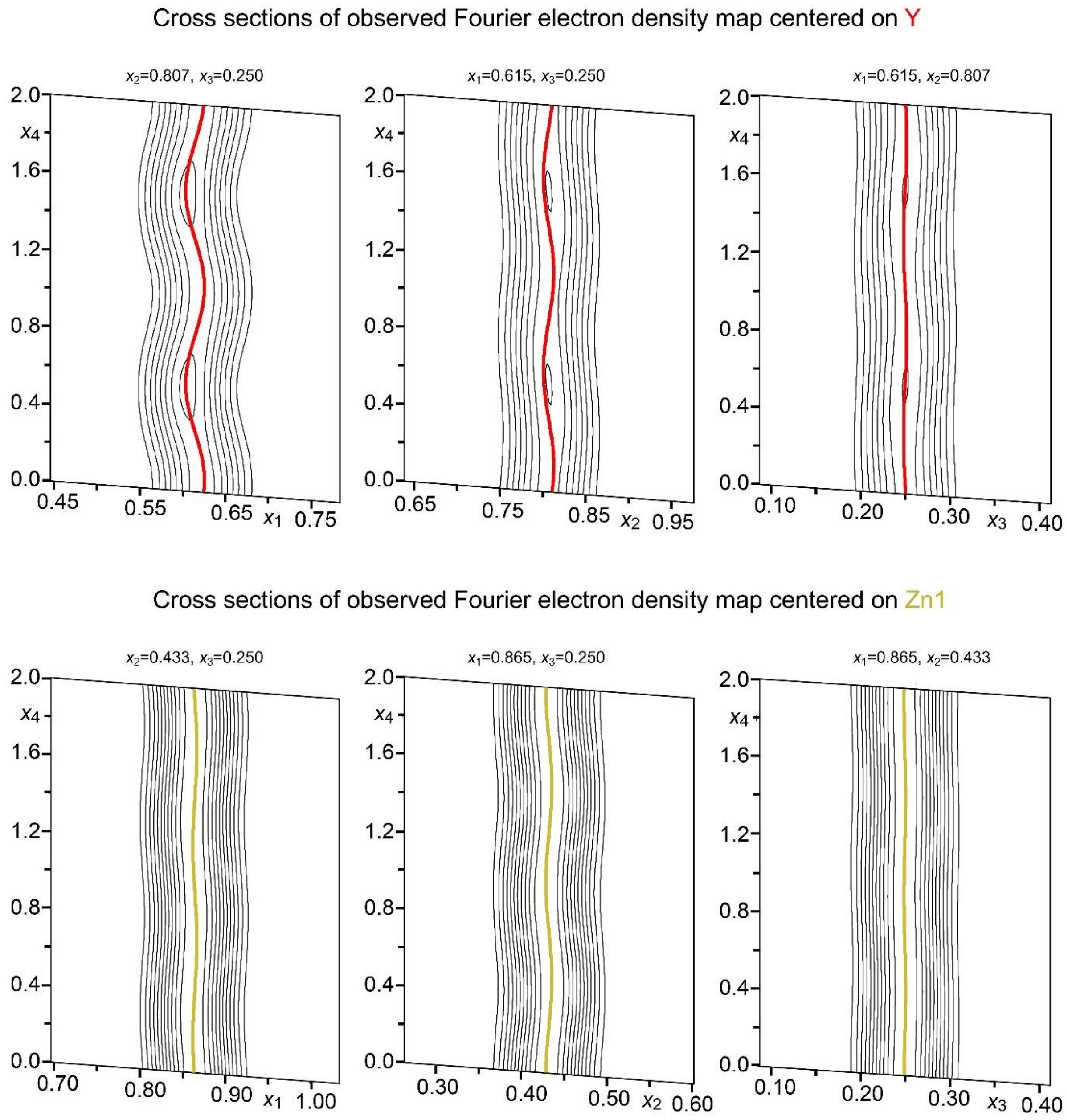

Cross sections of observed Fourier electron density map centered on Zn2

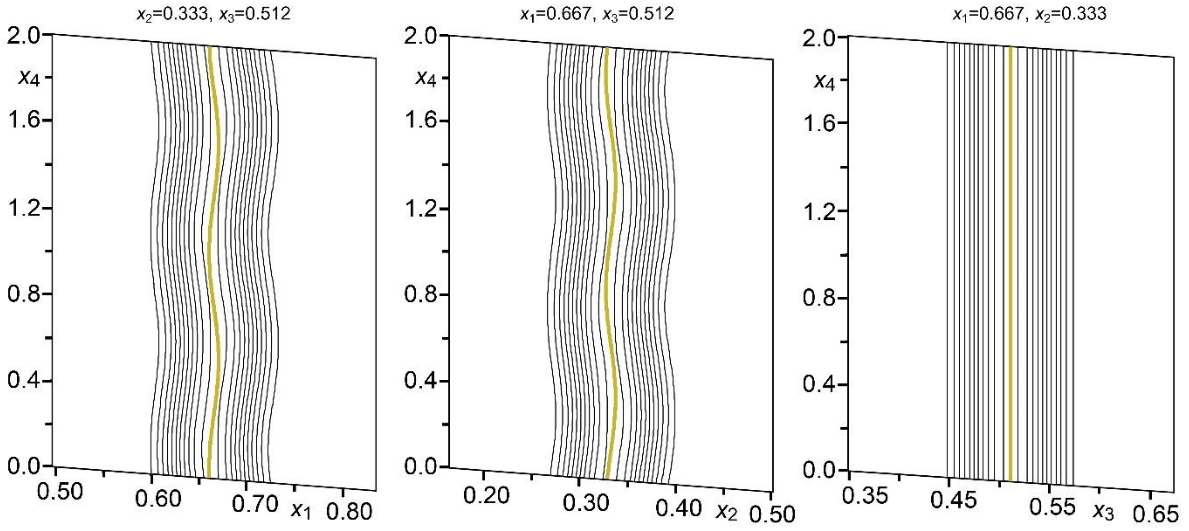

Cross sections of observed Fourier electron density map centered on Zn3

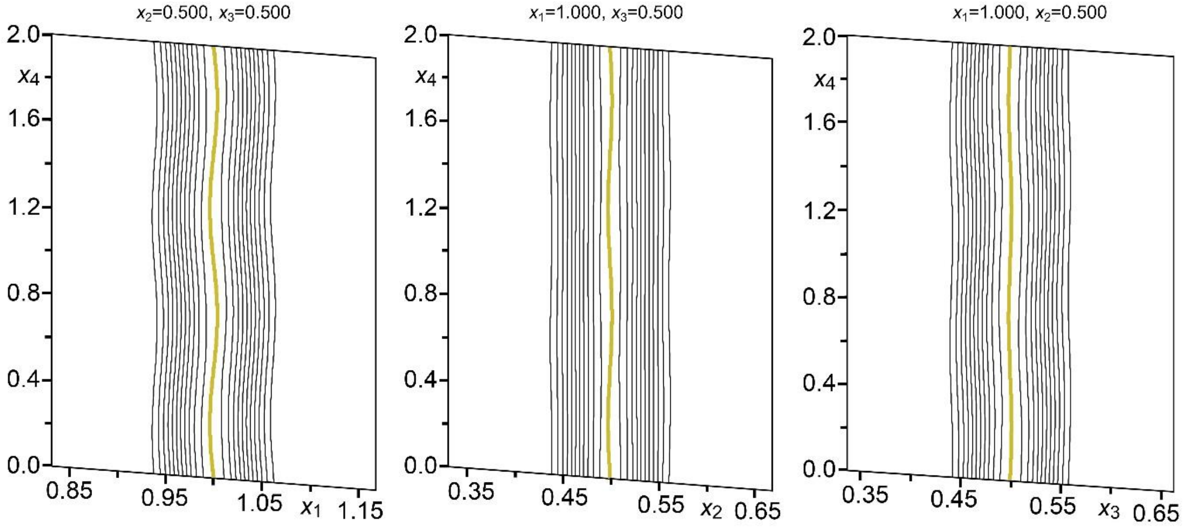

Cross sections of observed Fourier electron density map centered on Zn4

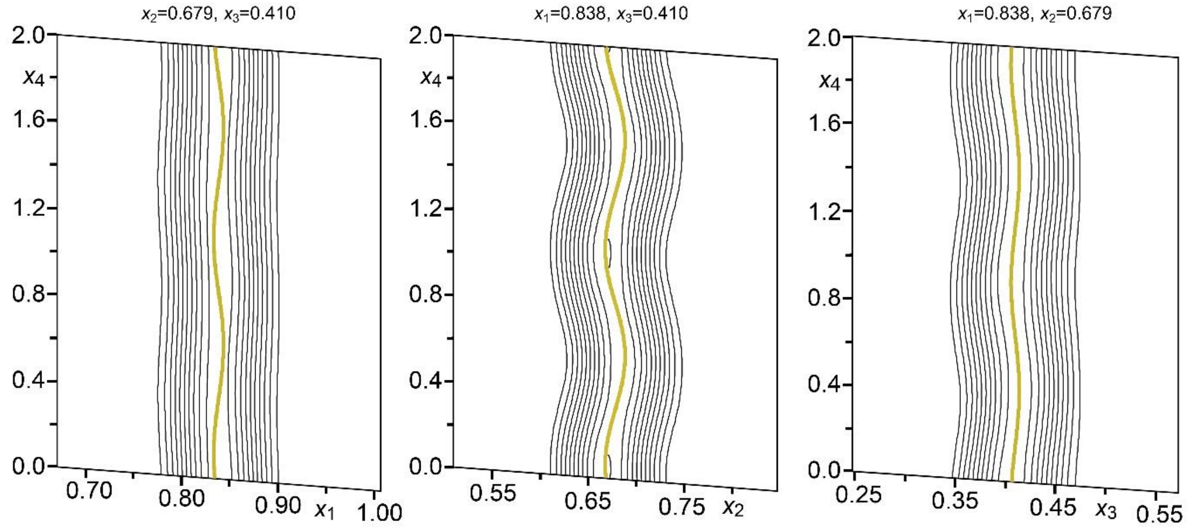

Cross sections of observed Fourier electron density map centered on Zn5

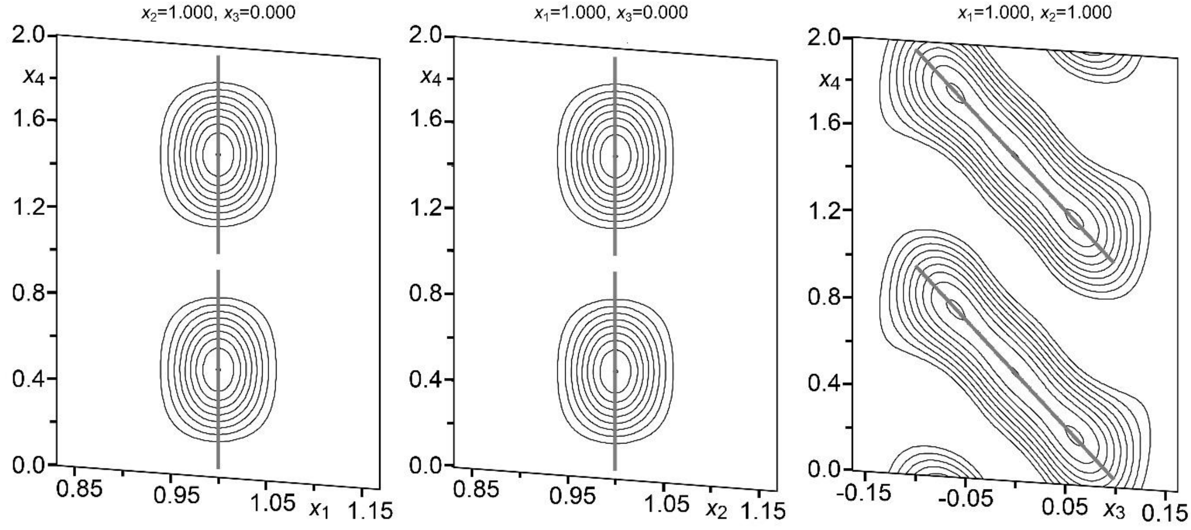

Cross sections of observed Fourier electron density map centered on Zn6

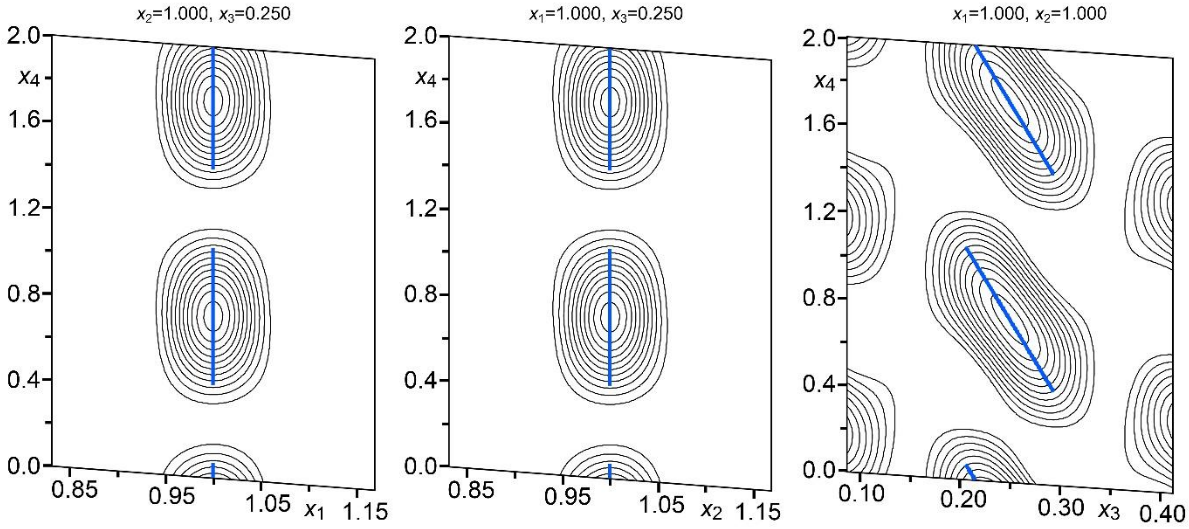

Figure S3 Examples of the YZn_{5+x} grains analyzed by EPMA. The lighter phase corresponds to YZn_{5+x} phase. (a) Line No. 86-92 (b) No. 136-140 in the lighter phase were used for WDS elemental analysis.

(a)

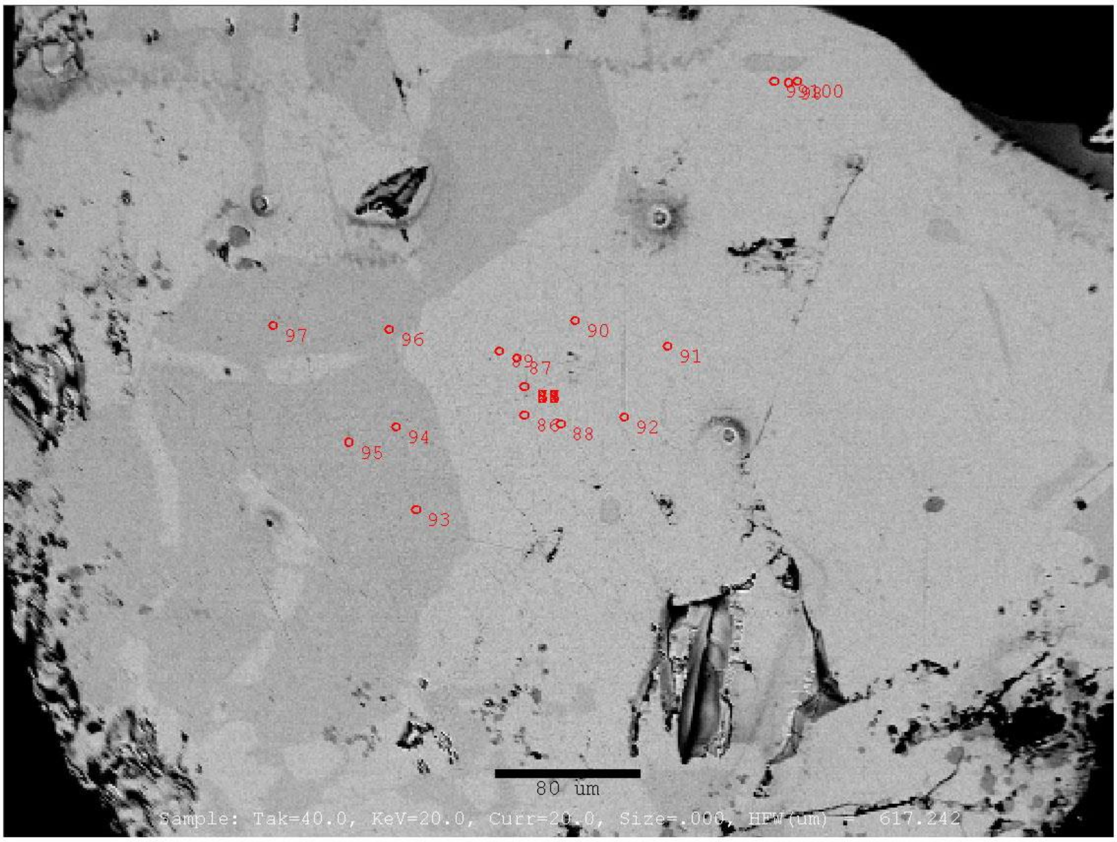

(b)

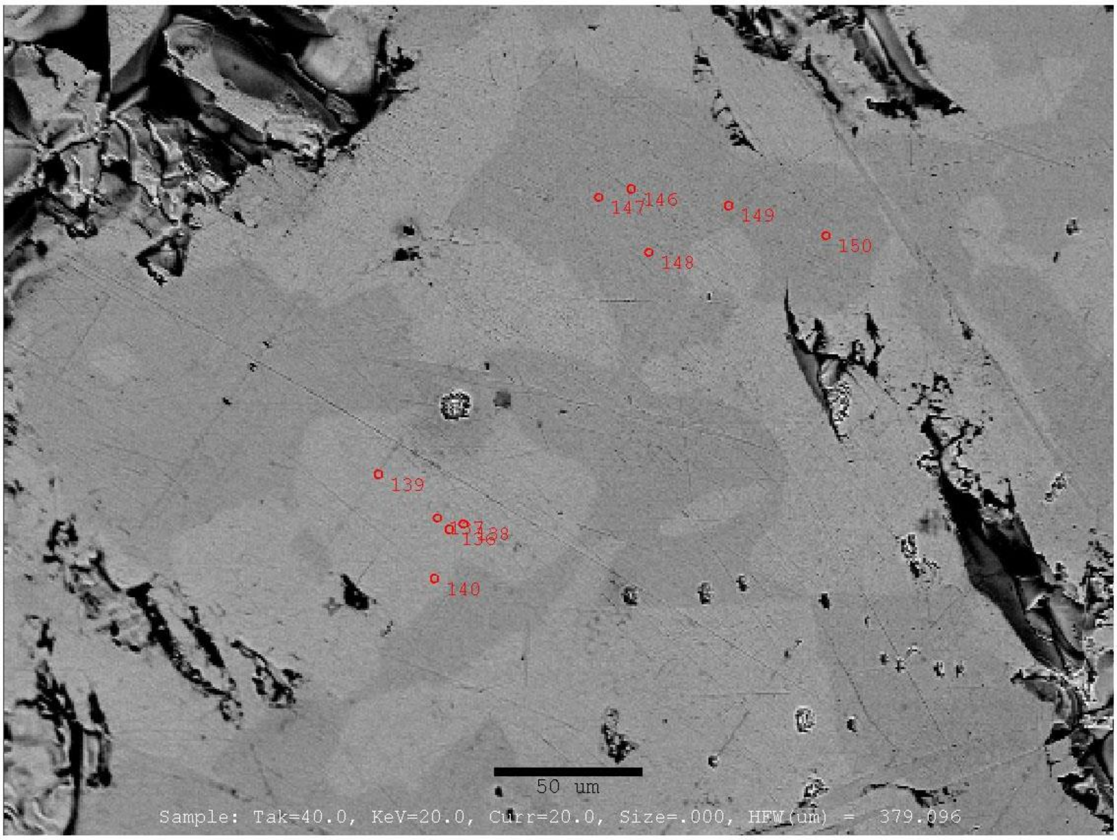
147
146
149
150
148
139
137
138
136
140
50 um
Sample: Tak=40.0, KeV=20.0, Curr=20.0, Size=.000, HFW(um) = 379.096
